# Developmental transcriptomics of the brittle star *Amphiura filiformis* reveals gene regulatory network rewiring in echinoderm larval skeleton evolution

**DOI:** 10.1186/s13059-018-1402-8

**Published:** 2018-02-28

**Authors:** David V. Dylus, Anna Czarkwiani, Liisa M. Blowes, Maurice R. Elphick, Paola Oliveri

**Affiliations:** 1 Research Department of Genetics, Evolution and Environment, University College London, Darwin Building, Gower Street, London, WC1E 6BT UK; 2 CoMPLEX/SysBio, UCL, Gower Street, London, WC1E 6BT UK; 3 School of Biological and Chemical Sciences, Queen Mary University of London, Mile End Road, London, E1 4NS UK; 4 Centre for Life’s Origins and Evolution (CLOE), UCL, Gower Street, London, WC1E 6BT UK; 5 Present address: Department of Computational Biology, UNIL, Genopode, 1005 Lausanne, Switzerland; 6 Present address: Centre for Cell Biology & Cutaneous Research, Blizard Institute, Barts and the London School of Medicine and Dentistry, Queen Mary University of London, London, E1 2AT UK

**Keywords:** RNA-seq, Endoskeleton, Biomineralization, Transcription factors, Regulatory states

## Abstract

**Background:**

Amongst the echinoderms the class Ophiuroidea is of particular interest for its phylogenetic position, ecological importance and developmental and regenerative biology. However, compared to other echinoderms, notably echinoids (sea urchins), relatively little is known about developmental changes in gene expression in ophiuroids. To address this issue, we have generated and assembled a large RNAseq data set of four key stages of development in the brittle star *Amphiura filiformis* and a *de novo* reference transcriptome of comparable quality to that of a model echinoderm—the sea urchin *Strongylocentrotus purpuratus*. Furthermore, we provide access to the new data via a web interface: http://www.echinonet.eu/shiny/Amphiura_filiformis/.

**Results:**

We have identified highly conserved genes associated with the development of a biomineralised skeleton. We also identify important class-specific characters, including the independent duplication of the *msp130* class of genes in different echinoderm classes and the unique occurrence of spicule matrix (sm) genes in echinoids. Using a new quantification pipeline for our *de novo* transcriptome, validated with other methodologies, we find major differences between brittle stars and sea urchins in the temporal expression of many transcription factor genes. This divergence in developmental regulatory states is more evident in early stages of development when cell specification begins, rather than when cells initiate differentiation.

**Conclusions:**

Our findings indicate that there has been a high degree of gene regulatory network rewiring and clade-specific gene duplication, supporting the hypothesis of a convergent evolution of larval skeleton development in echinoderms.

**Electronic supplementary material:**

The online version of this article (10.1186/s13059-018-1402-8) contains supplementary material, which is available to authorized users.

## Background

A fundamental question in evolutionary biology is how complex characters originate. Complex structures, such as the endoskeleton, sensory organs or central nervous system, are built during animal development and encoded by a precise program(s) that requires coordinated expression of many genes regulated by large networks. A comprehensive theory formulated a decade ago by Davidson and Erwin [1] explains both macro and micro evolutionary transitions as changes in gene regulatory networks (GRN) or rewiring. Therefore, comparative studies of gene expression during development have been used fruitfully in locating GRN rewiring that occurred during evolution [2].

The calcite endoskeleton of echinoderms provides an ideal system to study the evolution of complex characters at the level of GRNs. The phylum Echinodermata comprises five extant classes with well-supported phylogenetic relationships, with echinoids (sea urchins) and holothuroids (sea cucumbers) (Echinozoa) forming a sistergroup to asteroids (sea stars) and ophiuroids (brittle stars) (Asterozoa), and crinoids (sea lilies) as an outgroup [3–5]. While all echinoderms have calcitic skeleton as adults, only ophiuroids and echinoids develop an elaborate skeleton as larvae. In contrast, the larvae of the other three classes either develop only small ossicle primordia, called spicules (holothuroids), or do not form a skeleton at all [6, 7]. This provides an ideal evolutionary context to study the appearance and/or reduction/loss of complex morphological characters. The most comprehensive GRN model so far studied for an animal describes the development of the larval skeleton in the sea urchin *Strongylocentrotus purpuratus* [8–10]. It explains how in the course of development dozens of regulatory genes act together to specify a mesodermal cell population, which later form two ventro-lateral clusters on each side of the primitive gut (archenteron) and finally secrete the calcitic endoskeleton typical of the sea urchin pluteus larva (reviewed in [7]). Interestingly, whereas around 30 transcription factors (TFs) and a few signalling pathways are sufficient for the initiation, progression and maintenance of this process [10], more than 800 genes participate in the final step of cell differentiation and biomineralization of organic matrix. These differentiation genes have been identified using transcriptomic and proteomic experimental strategies [9, 11–13], although their roles and GRN linkages are largely unexplored. The extensive level of detail of the sea urchin GRN underlying skeletogenesis provides a useful framework to address questions about the evolution of development mechanisms through comparison with other echinoderms. Expression data are already available for a few orthologs of sea urchin skeletogenic transcription factor genes that have been identified in representatives of all echinoderm classes except crinoids [6, 14–16]. However, there has been relatively little comparative analysis of genes involved in skeletal differentiation in echinoderms.

Recently, biological and evolutionary studies have been transformed by immense technological improvements in sequencing technology [17]. Relevant to this study, RNA sequencing is now an established technique that provides a practical and cheap alternative to whole genome sequencing [18] because it allows rapid advancements in molecular genetic analysis of organisms for which limited or no genomic data are available but which are of great interest from an evolutionary and/or developmental perspective. Importantly, RNA sequencing enables a global quantitative analysis of gene expression at specific stages of life and/or in particular tissues/organs. In this way it is possible to reconstruct the timeline of expression of each individual gene and determine the progression of regulatory states, which is a key first step when analysing gene regulatory networks [19].

The large amount of molecular genetic information in echinoids compared to other echinoderm classes can be attributed to the fact that sea urchins have been studied extensively for over 100 years. Furthermore, the genome of the sea urchin *Strongylocentrotus purpuratus* was sequenced 12 years ago [20] and together with several improvements and additional mRNA sequencing data provides a very high quality resource [21, 22]. So far within the echinoderms, only the *S. purpuratus* genomic resources are of a high standard, although many additional species have been sequenced at lower quality [23]. Very recently the genome sequence of the Indo-Pacific sea star *Acanthaster planci* was published [24]. Furthermore, transcriptomic data are available for several echinoderm species, but with significant variation in sequencing depth and quality and with most datasets limited to a single life-stage or tissue [2, 25, 26].

Within the echinoderms, the brittle star class has received growing attention in recent years [27–30] due to their phylogenetic position as a sister group of sea stars, mode of development and regenerative capabilities. For instance, brittle stars develop a skeleton in the larvae similar to sea urchins [14, 31] and are thus a valuable model for addressing questions relating to differences and conservation of developmental genes involved in the formation of the larval skeleton. With this perspective, a single stage transcriptome identified many orthologs of sea urchin skeletogenic genes in a brittle star species [26], but no quantitative data on the dynamics of gene expression were provided. Furthermore, a comparison of skeletogenic regulatory states between an echinoid and an ophiuroid identified differences and similarities in the specification of the skeletogenic cell lineage [14]. Additionally, brittle stars regenerate their arms as part of their self-defence mechanism [32]. The re-development of the skeleton has been characterised in detail with respect to morphology and gene expression during various phases of regeneration [27–29, 33, 34]. Finally, brittle stars are used as important indicator species for ocean acidification studies [30].

Here we present a *de novo* transcriptome for the brittle star *A. filiformis* (Afi) obtained using four key stages of development, with the aim to provide a global quantitative assessment of developmental gene expression. We devised a computational strategy to generate a high-quality reference transcriptome, supported by several quality measures, and a reliable quantitative gene expression profile, validated on several candidates with other gene expression profile platforms, such as quantitative PCR and Nanostring. Focusing on the distinct feature of larval skeleton evolution within echinoderms, we assess the conservation of gene content by a large-scale comparison of our transcriptome with sequencing data from an asteroid, an echinoid, and a crinoid. Our results reveal a high-degree of conservation of genes associated with skeleton formation in the four species, consistent with the fact that all classes of echinoderms have a well-defined adult skeleton that originated at the base of the phylum. Contrary to previous studies, we identify major differences in the temporal expression of regulatory genes, which suggests a high degree of re-wiring for the developmental GRN. Furthermore, applying a fuzzy clustering approach, we find that most skeletogenic differentiation genes exhibit an increasing trajectory of expression during development, consistent with their hierarchical position as the final tier of a GRN. We also present an R-shiny application to allow access to all of the data presented here for future analysis.

## Results

### Assembly of a reference transcriptome for *A. filiformis*

**Fig. 1:**
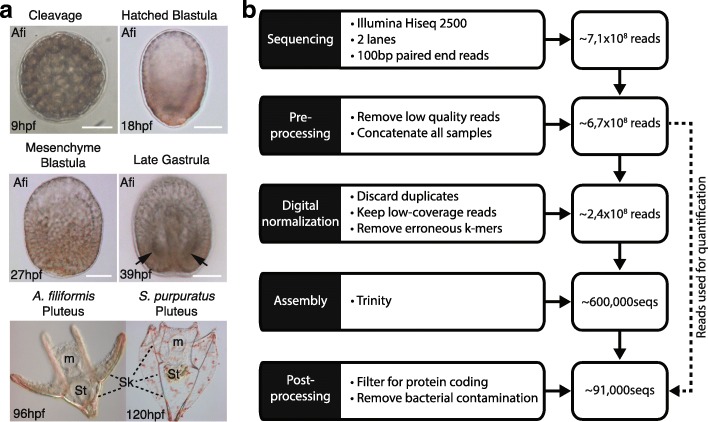
Pipeline used to obtain the *A. filiformis* developmental transcriptome. **a** Developmental timepoints used for RNA-seq: *9hpf* corresponds to a late cleavage stage, *18hpf* to a blastula stage, *27hpf* to a mesenchyme blastula stage and *39hpf* to a late gastrula stage (*arrows* point to position where spicules are formed). The brittle star *A. filiformis* and the sea urchin *S. purpuratus* pluteus larvae showing general morphological features and the birifrangent extended skeleton (*m* mouth, *St* stomach, *Sk* skeleton). **b** Assembly pipeline showing the individual steps and the reduction in sequences

Given the similarity of development between sea urchins and brittle stars [14, 31], we performed a global comparative analysis of the gene complement and gene expression profiles of representatives of these two classes of echinoderms. To enable this, we characterise for the first time the expression of genes in the brittle star *A. filiformis* using RNA-seq technology at four chosen key developmental stages that extend over the entire development of the larval skeleton, from early cell specification to final cell differentiation. The developmental stages are: end of cleavage stage (9 h post-fertilization (hpf)), a hatched blastula stage (18 hpf), three samples for mesenchyme blastula stage (27 hpf), and a late gastrula stage (39 hpf) (Fig. 1a). For the sequencing, we multiplexed the six samples using 100-bp paired-end reads on two lanes of Illumina HiSeq 2500, resulting in ~ 100 million reads per sample (Additional file 1: Figure S1 and Additional file 2: Table S1). We decided for this strategy to obtain a very high coverage of the different stage transcriptomes to reliably detect low expressed genes in the absence of a reference genome. Given our interest in protein-coding genes we used poly(A) selected fractions for sequencing. At the time of sequencing, Illumina HiSeq 2500 was the best sequencing platform available. Joining all the reads from each sequenced sample and following the khmer-protocols v0.84 [35], we assembled a reference transcriptome that would reflect all protein-coding genes expressed in the analysed stages (Fig. 1b). In this three-step assembly, we first trimmed all reads for Illumina adapters and low quality base pairs, then applied digital normalization to remove overrepresented reads and erroneous k-mers [36], and finally used the resulting reads as input for Trinity [37] (Additional file 2: Table S1). Our initial assembly resulted in 629,470 sequences. To determine whether the digital normalization step introduced artefacts, we assembled each individual sample omitting this step and compared them with the combined assembly. We recovered over 94 % of sequences using a BLASTn search (e-value 1E-20) of each individual assembly against the combined assembly (Additional file 1: Figure S2). Thus, we concluded that the digital normalization step did not introduce any significant bias in the combined assembly.

Because the focus of this study was on protein-coding transcripts, we filtered our initial combined assembly for all open reading frames that have an uninterrupted coding region longer than 300 bp (equivalent to 100 amino acids) using the TransDecoder package [38]. This reduced our dataset to 92,750 protein-coding sequences. We further removed any potential bacteria contaminates through application of a BLASTx search against 12,537,847 bacterial proteins (Uniprot DB; bacteria release 2014_06; 2563 species) and crosschecked the identified sequence for closer percentage of identity with hits obtained using a BLASTx (both e-value 1E-20) search against the Uniprot SwissProt DB (release 2014_07). Finally, we were left with 91,311 contigs constituting our reference transcriptome (RefTr; Table 1). The number of contigs produced by *de novo* transcriptome assemblers is typically large as assemblers cannot differentiate between isoforms or alternative transcripts of the same gene and thus report each separately (reviewed in Moreton et al. [39]). Moreover, artefacts such as repeats, sequencing errors, variation in coverage or genetic variation within a diploid individual create contigs that are not truly representative of different isoforms. As a result, transcriptome assemblers often report repeated contigs that differ only by a single nucleotide polymorphism (SNP), indel or fragmented versions of a transcript (reviewed in [39]). Moreover, simulation studies using error-free reads showed that *de novo* assemblers inevitably produce multiple contigs for the same gene [40]. To account for this type of variation in the absence of a reference genome, but without losing sequences, we partitioned similar contigs that differ due to SNPs or indels into transcript families that share a protein identity of at least 97 %. On average this approach grouped 1.3 contigs to each transcript family, resulting in 67,945 total transcript families. Unfortunately, splice variants and other artefacts are not incorporated into this type of clustering, leading to a number still larger than expected when comparing with the gene set of the sea urchin *S. purpuratus* gene set (~ 21,000 [21]), the only echinoderm for which high quality genome sequence data were available when this study was conducted. In the absence of an *A. filiformis* reference genome and so as not to bias the analysis, we chose to use the RefTr for further steps.

**Table 1:** Summary of quality statistics for the transcriptomic and genomic dataset used

Species	N25	N50	N75	Longest	Mean	Median	Shortest	Number of contigs
*Strongyloncetrotus purpuratus*	6297	4108	2438	22,850	2821	2139	400	29,072
*Amphiura filiformis*	2601	1410	639	18,993	925	525	297	91,311
*Patiria miniata*	1131	474	351	23,898	524	369	63	263,867
*Antedon meditarrenea*	1508	443	186	36,836	302	156	100	607,455

To test the quality of our assembly, we compared our RefTr with 48 isolated clones containing coding (cumulative length of 32,769 bp) and UTR regions (cumulative length of 7091 bp) sequenced using Sanger sequencing technology. Using BLASTn and collecting only the top hits, we obtained an average percentage of identity of 98.6 %. On an average alignment length of 588 bp we found ~ 7 mismatches in coding sequence, resulting in an average polymorphism in coding sequences of 1.2 %, a value to be expected based on the fact that clones were obtained from various batches of cDNA that are different from the samples used for the RefTr. In conclusion, we produced a high-quality reference transcriptome assembly that will provide a valuable resource for future studies in brittle star biology.

### Gene content of *A. filiformis* based on analysis of the developmental transcriptome

In order to have a meaningful comparative analysis of gene expression between brittle star and sea urchin clades, which diverged roughly 480 million years ago (mya) [5], we first classified and annotated the gene content of our RefTr and then assessed the evolutionary conservation of genes in the Echinodermata to better understand at a global level the conservation of genes and appearance of novel genes.

For this aim, and to be as comprehensive as possible, we applied independent search methods. First, we used the Blast2GO tool [41] that assigns gene ontology terms to each contig. Blast2GO first uses a BLASTx search (e-value 1e-3) against the GenBank non-redundant database and this search resulted in hits for 62,388 Afi contigs corresponding to 26,010 unique genes from 1334 different species. Consistent with ophiuroids being echinoderms, most hits were found for *S. purpuratus* (25,882/62,388 contigs), followed by the hemichordate *Saccoglossus kowalevskii* (Additional file 1: Figure S3). The second step of the Blast2GO pipeline performs an InterProScan to find regions within contigs that have conserved protein-coding domains. This step found 66,071 contigs with at least one region that has a recognizable protein domain. The combination of the BLASTx and interpro searches was then used to assign gene ontology terms, which provided functional classifications for 27,923 of our contigs (Additional file 1: Figure S3).

**Fig. 2:**
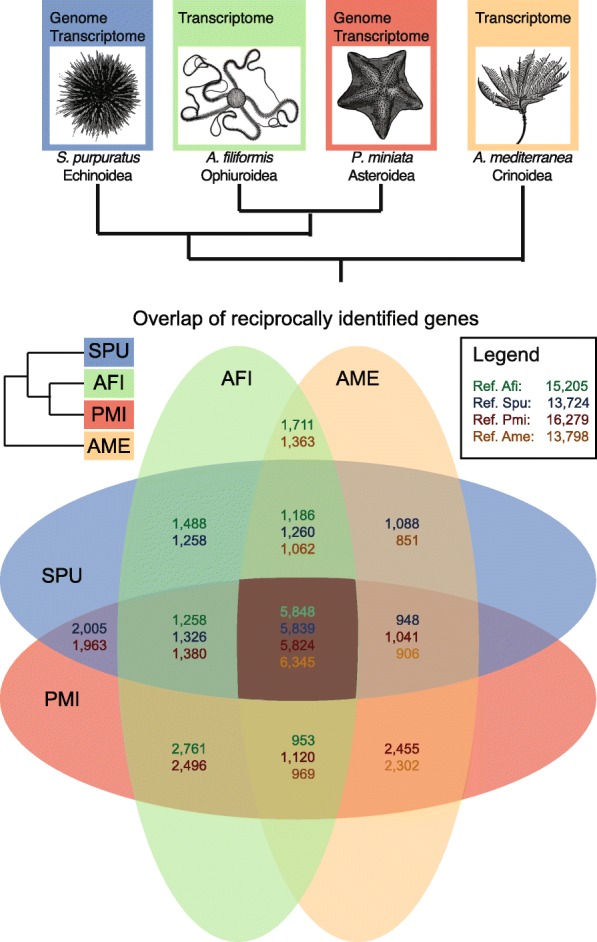
Gene content in representatives of four echinoderm classes. **a** Phylogenetic relationships of the four species compared in this study according to the currently most supported phylogeny for the classes these species belong to. **b** Venn diagram showing the overlaps of genes that were identified using a reciprocal tBLASTx (e-value 1e-6) strategy. The different numbers in each overlap field indicate the species that was used as reference for the BLAST search. *Afi Amphiura filiformis*, *Pmi Patiria miniata*, *Ame Antedon mediterranea*, *Spu Strongyloncetrotus prupuratus*, *Echi* Echinoderm core (overlap of all four classes)

To proceed with a general assessment of the evolution of gene content specifically in the Echinodermata, we collected in addition to the ophiuroid *A. filiformis* transcriptome (this study) representative datasets from the draft genome sequence of the asteroid *Patiria miniata* (Pmi; Baylor College of Medicine, HP081117-HP139664), the genome sequence of the euechinoid *S. purpuratus* (Spu) [20, 21] and the transcriptome of the skeleton-rich adult arm of the crinoid *Antedon mediterranea* (Ame) [42] (Fig. 2a). Differences in samples, sequencing technologies and assembly strategies make gene content comparisons from different species difficult. Therefore, we computed quantity and quality metrics, allowing us to make meaningful statements in relation to the properties of the individual datasets (Additional file 2: Tables S2, S3 and S4; Additional file 1: Figure S4). Importantly, at the time of the study only the sea urchin dataset had a well-curated genome and was improved by additional deep coverage transcriptome data [20, 21] and is thus used here as reference for comparative analysis. Our analysis indicated that all datasets are of comparable high quality (Additional file 2: Tables S2, S3 and S4; Additional file 1: Figure S4).

To gather information about the echinoderm-specific gene content we used a union of the Spu gene sets predicted from genome and transcriptome databases (29,072) to identify genes in Afi and the other echinoderm species by applying a tBLASTx (e-value 1e-6) search. For the identification we followed the khmer-protocols v0.84 [35]. In this protocol, a reciprocal BLAST is used on the sequences partitioned into transcript families. Reciprocally identified sequences are classified as orthologs and unidirectional identified sequences as homologs. Additionally, for contigs that are part of the same transcript family the BLAST result is propagated in order to ensure that the identification is consistent with the partition. Using this protocol, we found matches of Spu proteins for 41,492 of 91,311 of Afi RefTr sequences, for 77,576 of 263,867 of Pmi genome and transcriptome derived contigs, but for only 26,997 of 607,454 of Ame transcriptome-derived contigs. Detailed numbers are presented in Table 2. Importantly, the largest number of unique homologs of sea urchin proteins were identified in Pmi (16,211), followed by Afi (13,656) and Ame (12,982). This finding is consistent with the fact that the Pmi dataset is a combination of contigs derived from both genomic and transcriptomic data, whereas the Afi and Ame datasets are derived solely from transcriptomes. As a positive control for our strategy, we searched the Spu dataset against itself and found 91 % (Table 2) of hits had an e-value less than 1e-6. The residual 9 % of protein-coding sequences are likely to be highly similar sequences, such as recently duplicated genes, different alleles or potentially wrongly annotated genes, which in general fail to give a clear unequivocal result using a BLAST alone approach.

**Table 2:** Homologs of sea urchin proteins in other echinoderms

Species	Source	E-value	Reciprocal BLAST	Single BLAST	Number of unique Spu sequences	Number of qQuery sequences
*A. filiformis*	Transcriptome	1E-06	9779	41,492	13,656	91,311
*P. miniata*	Genome + transcriptome	1E-06	10,208	77,576	16,211	263,867
*A. mediterranea*	Transcriptome	1E-06	9164	26,997	12,982	607,454
*S. purpuratus*	Genome + transcriptome	1E-06	26,395	2675	26,475	29,072

To determine the extent of sequence conservation in the echinoderm phylum we computed the overlap of contigs shared between species. Therefore, we searched reciprocally all versus all species (tBLASTx, evalue 1E-6) using each time one of the four species as a reference (Fig. 2b). Our analysis shows that around 6000 sequences are common to all species analysed, corresponding to 25 % of the protein-coding sequences of the sea urchin reference species. Any other combination of two to three species identified at least 1000–2000 shared genes. This suggests that in each class a specific subset of ancestral genes has been retained and consequently that others have been lost or have diverged beyond recognition with the methods employed here. Notably, we observed a higher number of genes to be shared between Afi and Pmi compared to other pairs of species (Fig. 2b). This is consistent with the recently published phylogenetic analysis of echinoderm relationships, in which sea stars and brittle stars are sister groups [3, 4]. To validate this result, we applied the orthology matrix algorithm (OMA) [43], which computes highly reliable groups of orthologous genes using the Smith-Waterman algorithm for sequence alignment. The set of orthologous genes obtained allowed us to clearly distinguish the differences in genes shared between species [43]. Using OMA, we observe a much higher conservation between Pmi and Afi than in any other overlap of two species, i.e. ~ 7000 orthologs compared to ~ 2000–4000 orthologs (Additional file 1: Figure S5). Moreover, the variation in the number of genes among species overlaps indicates a highly dynamic evolutionary history in terms of gene conservation in the four classes of echinoderms analysed here. This is supported by the similar number of genes shared between two species and can be explained by the separation of the four classes early on in echinoderm evolutionary history (542–479 mya) followed by long periods of independent evolution [5, 44].

### Functional characterisation of echinoderm genes reveals conservation of a regulatory toolkit in echinoderms

**Fig. 3:**
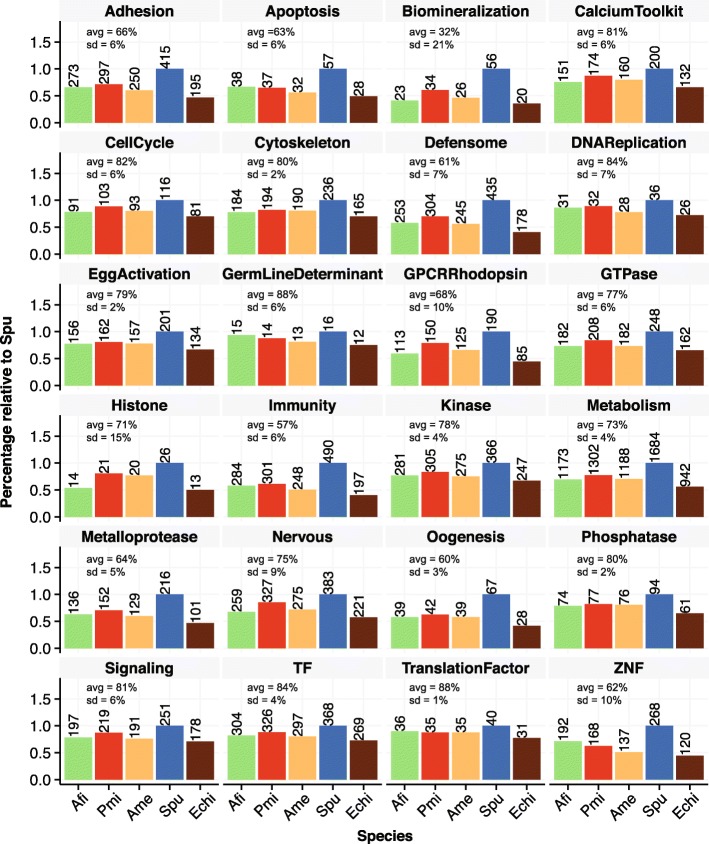
Conservation of gene functional classes in echinoderms. Sea urchin functional classes are based on *S. purpuratus* [21] and show proportions identified in the other three echinoderms. Average and standard deviation are calculated between Afi, Pmi and Ame and are normalised based on the sea urchin. *Afi Amphiura filiformis*, *Pmi Patiria miniata*, *Ame Antedon mediterranea*, *Spu Strongylocentrotus prupuratus*, *Echi* Echinoderm core (overlap of all four classes)

A recent study explored in detail a developmental transcriptome of *S. purpuratus* in terms of gene content and established echinoderm-specific ontology classifications [21]. Our high quality RefTr and consistent data treatment allowed us to apply this ontology classification and to compare the abundance of specific functional classes with other echinoderms. We queried our three species for the identified genes that belong to sea urchin functional classes (SUFC; Fig. 3). From a total of 6461 genes classified in 24 SUFCs we found 4494 homologs in Afi, 4407 in Ame, and 4976 in Pmi. We classified the SUFCs in three categories of conservation using manually selected thresholds. In the first category of highly conserved SUFCs (avg(Afi, Pmi, Ame) > 80 % of identified Spu sequences), we find Cytoskeleton, Phosphatase, Signaling, CalciumToolkit, CellCycle, TF, DNAReplication, GermLineDeterminant and TranslationFactorTF (Fig. 3). SUFCs that are conserved at a lower level (intermediate; avg(Afi, Pmi, Ame) between 70 and 80 % of identified Spu sequences) are Histone, Metabolism, Nervous, GTPase, Kinase and EggActivation; the lowest conservation of SUFCs (avg(Afi, Pmi, Ame) < 70 % of identified Spu sequences) is observed for Biomineralization, Immunity, Oogenesis, Defensome, ZNF, Apoptosis, Metalloprotease, Adhesion and GPCR-Rhodopsin (Fig. 3). Interestingly, Biomineralization, GPCR-Rhodopsin, Histones and ZNF show the highest level of variation between the three species (standard deviation > 10 %) and we find a high number of ZNFs only in brittle stars (Fig. 3).

To obtain a better picture of the conservation of the developmental program in general and the evolution of the larval skeleton in particular, we focused our analysis on regulatory genes (TF and Signalling) and on biomineralization differentiation genes. Out of 368 sea urchin TF genes, we identified 304 in the brittle star, 297 in the crinoid and 326 in the sea star. The 304 TF genes in the brittle star correspond to 82 % of the sea urchin TFs and represent the cohort of TF used in this species throughout development, a number comparable to estimates obtained for sea urchin development (~ 80 % of 283 TFs are expressed by late gastrula [45]). Consistent with the fact that the sea star dataset is a combination of genome and transcriptome, we find the largest number of homologs of sea urchin TFs (326) in this class of echinoderms. On the contrary, the lowest degree of conservation was observed in the crinoid (297 out of 368), which might be attributable to the fact that the Ame transcriptome was obtained from a single adult structure (the arm), although arms are formed from multiple tissue types. Generally, a similar degree of conservation was observed for signalling molecules (~ 76–87 %), but with more variation between Pmi, Ame and Afi (Fig. 3). The high level of TF and signalling conservation indicates that echinoderms share a similar regulome.

The biomineralization SUFC shows a higher degree of variation and we find generally less genes (~ 41–60 %), or a lower percentage of conservation. Interestingly, when looking more thoroughly in the biomineralization class of genes, of the 14 spicule matrix (sm) genes, only one gene in Afi seemed to be expressed at stages when the skeleton is developing and only one gene was identified in the Pmi genome and transcriptome sequences, indicating that the sm class of genes is quite small in the Asteroidea and quite inactive during larval skeletogenesis in the Ophiuroidea, by comparison with the Echinoidea. Homologs of more than 50 % of Spu genes belonging to the collagen, cyclophilin and carbonic anhydrase categories (Additional file 2: Table S5) were found in all species. Interestingly, in a first assessment we found few homologs of the nine Spu msp130 genes in the species analysed here (two sequences in Afi, three in Pmi and four in Ame), although many contigs showed sequence matches. Therefore, we investigated if there are actually more msp130 genes in the other species than the BLAST algorithm alone is able to discriminate. Using 18 candidate genes, we generated a multiple sequence alignment and built a hidden Markov model (http://hmmer.org, version 3.1b) in order to scan for other contigs with a msp130 signature. With this approach, we found several candidates in our dataset that had this signature but were different in terms of their amino acid sequence. In order to investigate their relation to the sea urchin msp130 genes we built phylogenetic trees using Bayesian and maximum likelihood methods, also including genes found in outgroup species. Our trees support class-specific duplications of msp130 genes, as displayed by their independent expansions in different branches of the tree (Additional file 1: Figure S6). This analysis suggests that while all echinoderms share a similar regulome, defined as the cohort of all TF and signalling genes encoded in a genome, some classes of sea urchin biomineralization genes are either absent or duplicated independently when compared to the other three species analysed here.

### Skeletogenic genes are conserved within the echinoderms

**Fig. 4:**
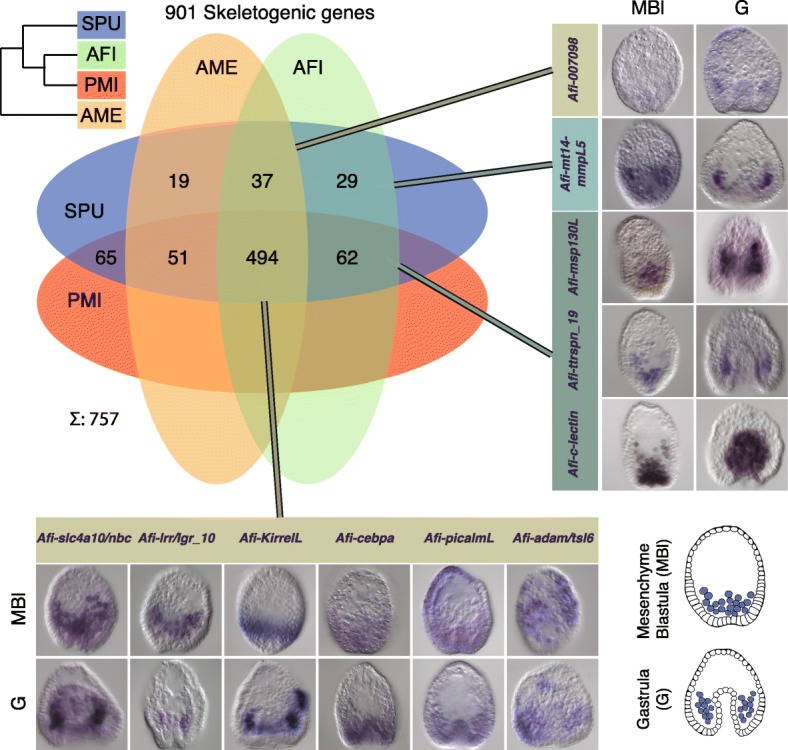
Homologs of sea urchin skeletogenic genes identified in other echinoderms and expression patterns for selected candidates. Venn diagram showing the overlap of genes involved in sea urchin skeletogenesis with homologs found in other echinoderms; 494/901 are shared between four classes of echinoderms, which is a higher proportion than a set of random genes (Additional file 1: Figure S7). Whole mount *in situ* expression patterns in two important brittle star developmental stages for several selected candidates from different regions of overlap reveals an association with cells associated with skeleton formation. In the *top right corner* is depicted the currently most supported phylogeny for these four species. Schematics representing mesenchyme blastula and early gastrula stages are in the *bottom right corner* (in *purple* are shown the mesenchymal cells that will give rise to skeleton). *Afi Amphiura filiformis*, *Pmi Patiria miniata*, *Ame Antedon mediterranea*, *Spu Strongylocentrotus prupuratus*, *Echi* Echinoderm core (overlap of all four classes). *MBl* mesenchyme blastula, *G* gastrula

All echinoderms develop a calcite skeleton and hundreds of genes are involved in this process. However, the SUFCs in the sea urchin include only 56 genes that are classified as biomineralization genes. To obtain a more precise picture of genes involved in skeletogenesis and their evolution we gathered 1006 sea urchin skeletogenic candidates based on literature searches. This extended candidate list was compiled from proteomic studies based on skeletal elements obtained from adults and larvae [12], a differential analysis of sea urchin mesenchyme blastula where skeletogenic mesenchymal cells were removed [9] or isolated [13] and a large scale morpholino analysis [10]; it is therefore representative of the skeleton developmental process from cell specification up to the deposition of the biomineralised skeleton. We updated this list with the latest annotation of the sea urchin genome and obtained 901 genes (Additional file 3). Of these 901 candidates, 37 are TFs and 32 are signalling molecules belonging to five different pathways (*i.e.*, Fgf, Vegf, Delta/Notch, Wnt and BMP), whilst the rest of the genes belong to various classes of C-type lectin-type domain, carbonic anhydrases, matrix metalloproteases, known skeletogenic matrix genes (sm and msp130) and others. To maintain a very broad view, we searched the homologs of our annotated species for these candidates with the aim to find a core set of skeletogenic genes and possibly a set specifically used in the development of the larval skeleton in echinoids and ophiuroids. We found 601 candidate skeletogenic genes in Ame*,* 622 in Afi and 672 in Pmi out of 901 genes in Spu, which follow a trend similar to the whole gene set. To display the differences in skeletogenic gene conservation we computed the overlaps between the four species (Fig. 4). Due to the fact that skeletogenesis in the adult is a feature present in the common ancestor of extant echinoderms, we wanted to check whether the 494 skeletogenic genes found in all four species are more highly conserved than a set of randomly selected genes. Therefore, we computed the overlap of 901 genes selected randomly 1000 times and compared it with the skeletogenic gene set (Additional file 1: Figure S7). Our analysis indicated that genes associated with the skeletogenic process are more conserved than a set of random genes (compare 494/757 to 278/613, chi-squared proportion test *p* < 0.001; Fig. 4; Additional file 1: Figure S8). This is in line with the evolution of the biomineralised ossicle in the form of stereoms at the base of the echinoderms and a high level of conservation of this structure throughout evolution. Although, this analysis gives us a good indication of the presence or absence of genes in the different classes of echinoderms, it does not provide evidence that these genes participate in skeleton formation. Recently, using a candidate approach we showed in a multi-gene expression study that of 13 TFs involved in Spu skeletogenesis 10 are active in Afi development, whilst the other three, although expressed during development, are not localised in cells giving rise to skeleton [14]. This highlights the importance of complementing transcriptomic data with spatial/temporal analysis of gene expression. Therefore, we selected from our list of 622 skeletogenic homologs 11 candidates of the differentiation cascade to investigate if they are expressed in the skeletogenic mesoderm (SM) lineage in brittle stars (Fig. 4). We found that all of these genes are either expressed specifically or are enriched in skeleton-associated cells during the development of *A. filiformis*. Most of them seem to be specifically enriched in the SM lineage at late gastrula stages in cells where the skeleton is deposited. Together with our previous analysis of developmental regulatory states [14], a total of 24 genes show expression in cells associated with biomineralised skeleton conserved in two distant clades: sea urchin and brittle star. This indicates a largely similar molecular make up of calcitic endoskeleton (65 %) in sea urchin and brittle star; and it is consistent with the ancient origin of the biomineralised skeleton in the form of stereom, which originated at the base of the phylum Echinodermata.

### A quantitative developmental transcriptome for *A. filiformis* to assess the dynamics of gene expression

Our prior analysis indicates that skeleton-forming genes are well conserved within the echinoderms, but what about the regulatory program? The developmental regulatory program is executed by a large GRN that tunes the expression of thousands of genes. To make an initial global assessment of the *A. filiformis* regulatory program we took advantage of the separate sequencing of four key developmental stages and the ability to obtain quantitative data from RNA-seq. While being relatively trivial to align reads when well curated gene models exist, this task is complicated for *de novo* assembled transcriptomes due to the high level of contig redundancy. In order to address this issue, we used the CORSET algorithm [46]. CORSET removes sequences with less than ten reads, which correspond to technical background level, and groups contigs to expression clusters (ECs) that share the same reads, thus resulting in expression values that are equivalent to potential gene counts. In a first step this algorithm removed 9854 sequences that were expressed with less than ten reads. The resulting 81,457 contigs were then clustered to 37,999 ECs (min 1seq, max 66seq, mean ∼ 2.1seq per cluster; Additional file 1: Figure S8). In order to normalise the dataset relative to an internal standard, we computed the standard deviation for each EC between the four time points and selected 331 ECs with standard deviation < 0.01 (a list of all ECs can be found in Additional file 4). We then divided the RPKM corresponding to each EC by the average of the 331 ECs and multiplied each by one million to normalise and to obtain EC counts in transcripts per million (tpm). Because of the grouping of contigs into ECs, the previous annotation could not be directly propagated. Therefore, we associated with each EC the most frequent annotation of its constituent contigs, giving orthologs priority over homologs. This caused a reduction from 13,656 to 11,695 uniquely found sea urchin sequences in Afi. Of the reciprocally identified sequences, only 350 were lost during this process, resulting in 9429 reciprocally identified sea urchin sequences. Possible reasons for this reduction are the filtering of a low level of expressed sequences (less than ten reads; see above) and contigs mapping to different genes in sea urchin actually belonging to a single one. A summary for losses mapped to the SUFCs is presented in Additional file 1: Figure S9. To estimate the quality of our approach we compared 29 genes quantified using qPCR and 86 genes quantified using Nanostring in different RNA batches with the corresponding ECs. We obtained a high correlation between qPCR and ECs (r2 = 0.84) and between Nanostring [47] and ECs (r2 = 0.77), supporting our quantification strategy (Additional file 1: Figures S10 and Figure S11). These quantitative data are now available for evaluating dynamicity of gene expression and comparative analysis and will be used for comparative gene expression with sea urchin.

### Temporal mode of TF expression in the brittle star shows many differences with the sea urchin

**Fig. 5:**
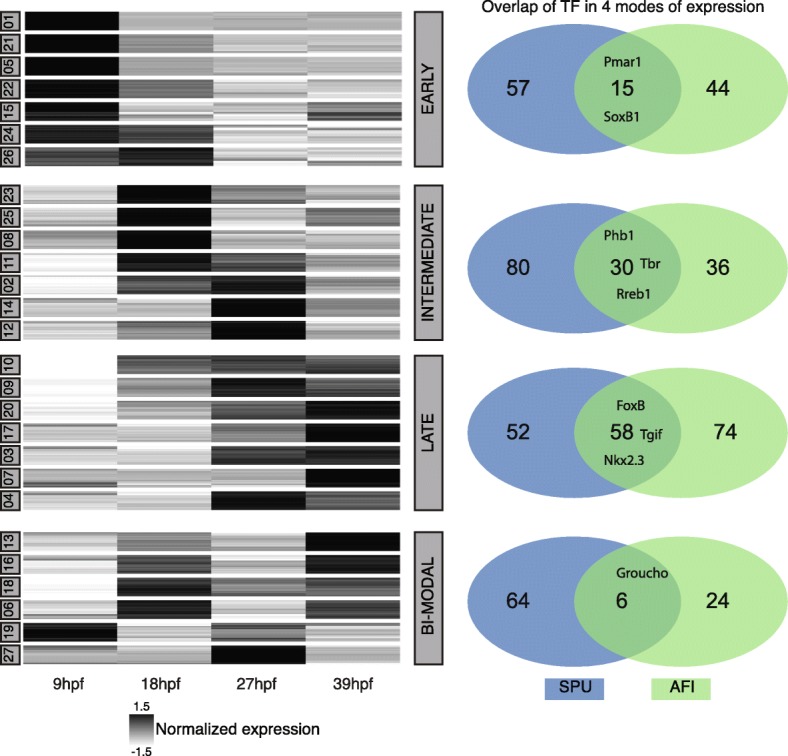
Global *A. filiformis* gene expression and comparison of larval regulatory states. **a** Fuzzy clustering of 39,000 ECs in 27 clusters of four developmental time points sorted in four distinct modes of expression (*EARLY*, *LATE*, *INTERMEDIATE*, *BI-MODAL*). Each line represents the expression of a single gene, and the *grey intensity* indicates the normalised expression. **b** Comparison of TFs in the four modes of expression between sea urchin (*SPU*) and brittle star (*AFI*). The majority of TFs show differences in expression

In order to obtain a global view of time-series expression during development and to group the genes by similar expression patterns, we applied a fuzzy clustering approach [48]. Based on the fact that between the four time points there are three possible modes of expression (no change, increase or decrease) we decided to assign to each EC one of 27 fuzzy clusters (FCs). This algorithm assigned 27 FCs to the 37,900 ECs. During this process 99 ECs were lost because they were not active throughout our four developmental time points but were expressed in one of the other two 27-hpf samples that were not used for this analysis. We re-iterated this algorithm 100 times and optimised the membership of each EC to a specific FC. A closer look into the 27 FC showed four distinct modes of dynamic behaviour and we decided to use this grouping for future analysis. The groups were EARLY with 10,593 FCs, INTERMEDIATE with 8531 FCs, LATE with 9968 FCs, and BI-MODAL with 8808 FCs (Fig. 5a). EARLY FCs contained ECs that showed decreasing expression across the first three time points and thus were likely to have a role during very early development (9 hpf, end of cleavage). In these FCs, we found genes that are responsible for early specification and are only transiently active. In total, we found 59/287 TFs and 105/561 skeletogenic genes that showed a decreasing trajectory over the four time points. In this group, only *Afi-pplx* was found as a gene involved in Afi skeleton specification. In the INTERMEDIATE group were genes whose expression trajectories peak at either 18 or 27 hpf and then decrease steadily. Examples of genes found in this group are *Afi-alx1*, *Afi-tbr*, *Afi-gataC* and *Afi-erg*, TFs that have been shown to be expressed in mesodermal cells of the Afi embryo and known to play a role in the specification of mesoderm [14]. In total, this group comprises 66/287 TFs and 68/561 skeletogenic genes. In order to form the extended larval skeleton, we expected most of the skeletogenic genes previously described to be expressed at the moment of the deposition of the calcite skeleton, and therefore to show an increasing pattern of gene expression. Indeed, most of the skeletogenic genes were clustered in the LATE group 287/561. Among others, this group contained the biomineralization genes *Afi-p19 (Cah10L)*, *Afi-p58a*, *Afi-p58b*, *Afi-ttrspn_19*, *Afi-slc4a10/nbc* and *Afi-c-lectin*, all expressed in skeletogenic cells in brittle star (Fig. 3) [14]. Moreover, the LATE group contained most of the active TFs (132/287), consistent with the increasing complexity of cell types over developmental time. The final group, called BI-MODAL, consists of two expression peaks throughout the four time points and contains 30/287 TFs and 101/561 skeletogenic genes. This group contains genes that might be expressed in different domains during development, potentially having two (or more) roles throughout development. Examples are *Afi-hesC* and *Afi-delta,* which are first expressed in the mesodermal cells at the vegetal side of the embryo at the blastula stage (18 hpf) and then in scattered cells in the ectoderm at the gastrula stage (39 hpf) and at the tip of the archenteron throughout gastrulation [14]. Based on the fact that our four time points correspond to four different stages of development, our grouping shows consistent activity of TFs involved in multiple stages of cell specification.

The direct output of a GRN is the temporal expression profile of each gene throughout time and each expression profile is linked to its regulatory state. Therefore, comparing temporal expression profiles between two species provides a first glimpse of GRN rewiring and heterochronic gene expression. In order to evaluate the differences and commonalities of TF usage between sea urchin and brittle star, we selected four time points that correspond to similar stages of development from the sea urchin transcriptome: they are 10, 18, 30, 40 hpf, in agreement with the comparative developmental stages previously described [14]. On these *S. purpuratus* transcriptome stages we performed a fuzzy clustering as for *A. filiformis*, and we then grouped the clusters based on the above-mentioned criteria. We identified in the EARLY category 72, the LATE 110, the INTERMEDIATE 110 and the BI-MODAL 70 out of the 368 TFs, and six genes are not classified due to too low levels of expression. When comparing TF expression, and therefore the developmental regulatory states between *S. purpuratus* and *A. filiformis,* many differences emerge in the four categories. In all four categories, we see more variation than overlap of TFs (Fig. 5b). For example, only 15 transcription factors in the EARLY category are in common between the two species (e.g. *pmar1* and *soxB1*), whereas 44 Spu homologs in Afi categorised as EARLY differ from the other 57 TFs in Spu expressed in this mode. Other examples of common transcription factors are for BI-MODAL *groucho*, for INTERMEDIATE *alx1, erg, foxM, mitf,* and for LATE *foxB, hnf4, tgif*. A summary of all TFs can be found in Additional file 5. This comparison highlights that TFs are used differently, or at least with a different timing of expression, during the development of the two species. Examples of such genes are *hesC* and *ets1/2*. Notably, there are more differences in the early phases of development when cell specification begins than in the late stages when cells initiate their final differentiation. Given that the direct output of a GRN is reflected in the temporal gene expression, this suggests differences in the topology of gene regulatory networks between Afi and Spu.

## Discussion

Here we present a *de novo* transcriptome of *A. filiformis* that samples four important stages of the embryonic development of this organism. We also present an overall strategy to effectively compare different data sets and to use RNA-seq quantitative data in the absence of a reference genome. Our data and assembly/annotation strategy are then used to obtain insights into two key evolutionary questions: how did the larval skeleton in echinoderms evolve and how conserved is the regulatory program of the pluteus larvae of sea urchins and brittle stars?

To assemble the *A. filiformis* RNA-seq data, we used a strategy with digital normalisation followed by application of the Trinity assembly. Our approach with digital normalisation allowed us to obtain a reference transcriptome that incorporated six independent samples within 4 weeks of computation on a server with only 64 GB of RAM, with quality comparable to assembly obtained with non-normalised data. Our comparison is in agreement with what was observed by Lowe et al. [49] for the assembly of sequence data from two closely related ascidians, for which a systematic comparison of assembly with and without digital normalisation showed no inclusion of computational artefacts, but a reduction of time and resources needed for the assembly. We show that our RefTr is of high-quality by various computational and experimental methods and we also applied the computational quality control to the other datasets to strengthen the subsequent comparative analyses. In the developmental transciptome the depth of sequencing (~ 100 million reads per sample) and the combination of samples from multiple stages were important driving factors that made such a high-quality assembly possible. Altogether our analysis shows that deep sequencing combined with a good pipeline can result in an assembly that is comparable to a genome in terms of gene capture. This is illustrated by the high number of genes that showed more than 90 % identity to genes in the Swissprot database. Thus, our transcriptome performed best when compared to other genome and transcriptome datasets (Additional file 1: Figure S4). Interestingly, our extraction of protein-coding genes reduced the total number of contigs from ~ 600,000 to ~ 90,000 (15 %), increasing the N50 value, but not affecting gene recovery, as shown in the CEGMA and BUSCO tests (Additional file 2: Tables S3 and S4). Based on our analysis only 15 % of the RefTr sequences are protein-coding, giving rise to a particular question: what are the residual 85 % of sequences? One possibility is that they are part of non-coding sequences (e.g. non-coding RNA, transcribed pseudogenes) or partially or wrongly assembled transcripts. Efforts to obtain genome sequence data for *A. filiformis* are underway to help obtain answers to these questions. Indeed, studies on human genomes show that more than 60 % of the genome is reproducibly represented in long RNA sequences, while only 2.9 % is represented by protein-coding sequences [50].

During the Cambrian period the rapid expansion of animal life was associated with acquisition of the capacity to form hard mineralised tissues, as testified by the first appearance of a fossil record for many phyla. Amongst others, echinoderms evolved their characteristic calcitic porous endoskeleton formed of magnesium-rich calcium carbonate and occluded proteins [51, 52]. A first step towards understanding the evolution and developmental genetics of a complex character such as a mineralised skeleton is to perform a comparative and phylogenetic analysis of gene content (Fig. 2). For this reason we compared four echinoderm classes, three of the Eleutherozoa subphylum (Echinoidea, Ophiuroidea and Asteroidea) and a crinoid outgroup, with a focus on the genes involved in skeleton formation. Studies on sea urchins have shown that several genes used during adult skeleton formation are also used in larval skeleton [12, 53], leading to the idea that an ancient regulative and differentiation module originated at the base of the phylum Echinodermata and then was secondarily co-opted to form larval skeleton. However, it is hotly debated whether this happened only once in the branch leading to the Eleutherozoa, or whether it occurred independently in both the sea urchin (Echinoidea) and brittlestar (Ophiroidea) lineages. The two transcriptomes used in this analysis correspond to stages (late gastrula, for *A. filiformis*) or structures (adult arm for *A. mediterranea*) in which the biomineralised skeleton has been deposited. Therefore, expression of genes involved in this process must be highly represented. It is important to clarify that due to the nature of this comparison, genome vs transcriptome, we can unequivocally evaluate only the gene (or protein-coding transcripts) present in at least two data set. On the other hand, the absence of genes in *A. filiformis* and *A. mediterranea* transcriptomes at stages or in structures with skeleton can be interpreted as lack of expression, suggesting that these genes are not used in building skeletal structures of these two organisms.

Our analysis revealed a gene toolkit of 494 genes conserved in all four echinoderm classes (Fig. 4), which potentially corresponds to the echinoderm core of skeletogenic genes. Indeed, our analysis of spatial expression shows that several of these genes are expressed in cells known to form the skeleton in the developing *A. filiformis* embryo (Fig. 4) [14] and a few of them are also known to be expressed during *A. filiformis* adult arm regeneration [29, 34]. Of the initial 901 gene set, only 37 are TFs and 32 signalling molecules. Of these regulatory genes, 84 % (58/69 regulatory genes) are conserved in all the echinoderm classes analysed, while only 52 % (436/832) of the other genes, which can generally be classified as differentiation genes, are conserved in all the classes, indicating a higher conservation of the skeletogenic cell regulatory program and a rapid evolution of echinoderm skeleton-forming genes. A closer look into these 436 genes using the sea urchin functional classes revealed that metalloproteases and biomineralization genes are actually the most variable class of genes (Additional file 1: Figure S9). This observation indicates that solely looking into these two categories can produce a biased picture of evolution, because only these two categories of differentiation genes showed a high level of variation and indicate low selective pressure. How can we explain the variation in the biomineralization genes? They are grouped in six categories, of which collagens, cyclophillins, carbonic anhydrases and an unnamed category [22], which include P16 [54] and other genes, are highly conserved in our selected representatives of the four classes of echinoderms. On the other hand, of these six categories, msp130 and spicule matrix (sm) genes show the highest level of variation. Indeed, of the nine sea urchin msp130 genes only two are found in all four species analysed (*Spu-Msp130r6* and *Spu-Msp130L*). An in-depth look into the brittle star transcriptome, using a hidden Markov model, revealed also the presence of seven other msp130 contigs that show differences at the amino acid level higher than the 1.2 % of polymorphism identified in the coding region, suggesting the presence of several genes. Indication that clade-specific expansions took place is strongly supported by our phylogenetic analysis (Additional file 1: Figure S6), which shows a consistent group of sea urchin *Msp130* genes with various paralogues represented in both sea urchin species analysed (*S. purpuratus* and *L. variegatus*), a different group of ophiuroid *Msp130s*, as well as other clade-specific expansions consistent with what has already been shown for *Msp130* genes in molluscs and annelids [55]. Concerning the spicule matrix (sm) genes, out of the 14 genes identified in sea urchin only the C-lectin that does not contain a proline-rich region is conserved in all four species. Therefore, no spicule matrix genes, characterised by a C-lectin domain and a conserved proline-rich domain [56], are found in any other class of echinoderm in stages when skeleton is built, making them likely to be a sea urchin-specific set of skeletogenic matrix genes. Further support for this hypothesis is provided by the following observations: First, a proteomic study of skeletal elements in another species of brittle star, *Ophiocoma wendtii*, did not find orthologs of these genes [16]; however, other potential candidates of c-lectin type genes for brittle star skeletogenesis were obtained, which are also present in our transcriptome of *A. filiformis* and which are expressed during larval and adult skeletogenesis [14, 34]*.* Second, in the *S. purpuratus* genome the sm genes are present in mini clusters of tandem repeated genes (Additional file 2: Table S7 and Additional file 1: Figure S12), suggesting a relatively recent duplication of these genes in the sea urchin lineage. Third, no such gene has been found in the hemichordate *Saccoglossus kowalevskii* genome [57], an outgroup of all echinoderms. Fourth, no spicule matrix genes have been found in the adult crinoid arm transcriptome analysed in this work (Additional file 2: Table S5). Both spicule matrix genes and msp130 genes have been highly duplicated in sea urchin, as seen in the many tandem duplications, and the presence of both in the pencil urchin *Eucidaris tribuloides* [23], indicating that this diversity had already evolved in the common ancestor of cidaroids and euechinoids. In this context, it would be interesting in future studies to analyse holothuroids as a sister class to the echinoids to pinpoint more exactly the evolutionary origin of this category of biomineralization genes. Interestingly, similar to these findings in echinoderms, the rapid parallel evolution in different lineages of genes associated with skeleton formation has also been reported for shell genes in molluscs and brachiopods [58, 59].

**Fig. 6:**
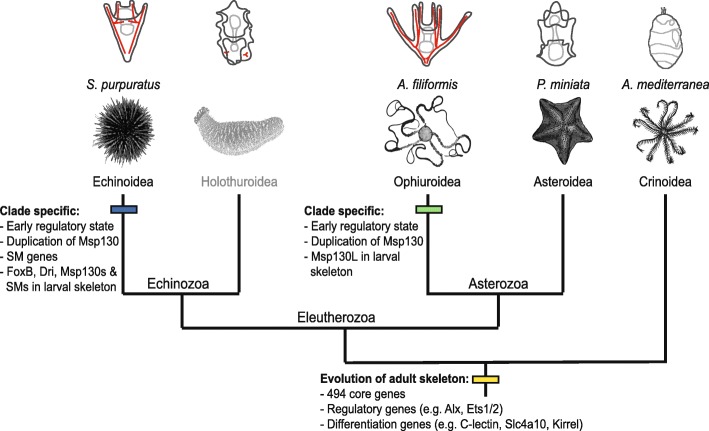
Scenario of larval skeleton evolution. A simplified phylogeny of echinoderms with representative larval stages (*skeleton in red*), which illustrates the position of major transitions in the evolution of the larval skeleton. Specifically, at the base of echinoderms are shown common features for the evolution of the adult skeleton and at the class level are depicted specific features for ophiuroids and echinoids

The fact that *msp130* and sm genes are expressed in both adult and larval skeletal structures in sea urchin [12] suggests that the evolution of sm genes in echinoids and the independent expansion of *msp130* genes occurred before the evolution of the echino-pluteus, the sea urchin larva with extended skeleton (Fig. 6). Similarly, in brittle stars *Afi-Msp130L* is expressed in the larval skeletogenic cells, supporting the argument that larval skeletogenesis evolved independently in the two lineages, potentially in both cases as a co-option of the adult skeletogenic program after clade-specific gene expansion took place. Other evidence in support of evolutionary divergence of the echinoid and ophiuroid pluteus larvae is provided by our comparative analysis of regulatory states in developing embryos (Figs. 5 and 6), defined as the sum of transcription factors expressed in a given cell at a given developmental time. We compared the transcription factor usage in *S. purpuratus* [21] with usage in *A. filiformis*, taking advantage of the quantitative aspects of transcriptome data and the sequence data from four key developmental stages: cleavage stage (9 hpf), when maternal mRNAs are still present and the zygotic genome starts to become active; blastula stage (18 hpf), when territories that will give rise to multiple cell types are specified and transcription factor genes are expressed in a spatially restricted manner [14]; mesenchyme blastula (27 hpf), when territories are further subdivided, cells continue in their specification pathway, and morphogenetic movements commence; and finally gastrula stage (39 hpf), when cell types are specified, morphogenetic movements are almost completed and cell differentiation is underway. This comparison shows that the early regulatory states, which determine the developmental GRN, of these two species are quite different. On the contrary, when cell types are specified and terminal selector genes (LATE genes in this analysis) are expressed [60], they show a similar regulatory make up in these two classes of echinoderms, suggesting extensive GRN rewiring in the early stages of development. Taken together, our findings are in agreement with the hypothesis that the peripheries of the GRN (i.e. early regulatory input and differentiation gene batteries) are the least constrained and thus the most frequently changed [1] part of a GRN, while the phylotypic stage (identified as the gastrula stage in echinoderms) [61, 62] is subject to strong evolutionary constraints. In this view our data support the idea that the regulatory states that define cell type identities, before differentiation, are the most evolutionarily stable compared to early specification regulatory states. In the case of the developmental program for echinoderm skeleton, this likely corresponds to the transcription factors conserved in all four classes analysed here and known to be expressed in skeletal cells [10, 14, 29]. Indeed the high degree of conservation in all four classes is consistent with all echinoderms forming an adult skeleton by similar ossicle units—the stereom [51]—and indicates that the GRN for adult skeletogenesis is a highly conserved feature. This is additionally supported by comparing expression patterns of several genes in juvenile or adult stages [29, 53, 63], which show a high degree of conservation in cells that participate in adult skeletogenesis. Additionally, in brittle star development most differentiation genes show an increasing trajectory over time, consistent with their role in the final differentiation of the biomineral structure.

The modelling of developmental GRNs requires knowledge of spatial and temporal expression. For a GRN analysis comprising a few genes, the integration of such data is a relatively simple task. In a systems biology perspective, however, where hundreds or thousands of genes are considered simultaneously, it is easy to lose track of the important details of a few or single genes, especially when working on novel systems with little to no access to the established data. Thus, we developed a website (http://www.echinonet.eu/shiny/Amphiura_filiformis/) using R-shiny that allows users to query different types of information, similar to that implemented by Tu and collaborators in 2014 for *S. purpuratus* [22]. Using the statistical programming language R as the backbone, our website provides a platform to easily query and find genes of interest. It gives access to annotations, expression levels, sequence information, differential screening and spatial expression patterns. Contigs can be queried by annotation, expression cluster id, contig id and additionally by the sea urchin functional classification. Thus, for example, one can easily retrieve all transcription factors sequences and their expression temporarily and spatially (where available). Moreover, spatial expression data can be extended by simply adding a folder with the contig id and the individual pictures as JPEG files. In future work, this website will be extended with data from regenerating arms produced in our laboratory and will thus create a unique resource to establish the brittle star *A. filiformis* as a developmental and regenerative model system.

## Conclusions

The data reported here show a large conservation of the genetic toolkit for echinoderm biomineralised tissues, and also highlight clade specific differences. By comparing gene expression profiles, we find major differences in temporal usage of TFs in early development, and clade specific gene duplication of important differentiation genes. These indicate a higher degree of rewiring at the periphery of the developmental regulatory network. Our study greatly influences the understanding of larval evolution and supports the hypothesis of convergent evolution of larval skeleton in echinoderms by independent co-option of a large GRN module underlying the development of the calcitic endoskeleton.

## Methods

### Experimental techniques

#### Embryological techniques

*A. filiformis* cultures were set up as previously described [14]. At the desired stage, embryos were collected for RNA extraction and/or fixed for WMISH as described in [14].

#### Cloning and probe synthesis

All genes used for spatial expression analysis by whole mount in situ hybridization (WMISH) were PCR amplified from *A. filiformis* cDNA and cloned in pGEM-T easy vector system (Promega) or Topo PCR cloning system (Invitrogen) according to the manufacturer’s instructions. Antisense probes labelled with DIG (Roche) were synthesised as previously described [14]. Primers are presented in Additional file 2: Table S5.

#### Quantitative PCR

qPCR was performed on different biological replicates to those used for the mRNA-seq, employing the procedures described previously [14].

#### Whole mount in situ hybridization

Spatial expression of selected genes at mesenchyme blastula (24 and 27 hpf) were characterised using WMISH as previously described [14].

#### RNA extraction

For mRNA sequencing, embryo samples of a single male and single female culture were collected at 9, 18, 27 and 39 hpf. At 27 hpf three samples were collected, two of which were chemically perturbed. The RNA extraction was performed as previously described [14]. The quality of extraction and concentrations were checked using NanoDrop 2000 and Bioanalyser.

#### mRNA sequencing

Sequencing libraries were prepared using the TruSeq RNA library preparation protocol. The samples were sequenced with Illumina v3 chemistry using the multiplex paired-end sequencing protocol. The sequencing was performed on an Illumina HiSEQ 2500 with 100-bp paired-end reads. To reach optimal coverage we sequenced two lines multiplexing the six samples. Library preparation and sequencing were performed at the SickKids Hospital, Toronto, Canada.

### Computational procedures

If not otherwise stated, all computational work was performed on an Apple Mac OS X 10.6 server with 24 cores and 64 GB of memory.

#### Assembly

The assembly pipeline and annotation followed a set of unified protocols described in [35]. The obtained reads were trimmed for adapters and for low quality sequences using Trimmomatic v0.27 (ILLUMINACLIP:Adapters.fasta:2:30:10; HEADCROP:12) [64]. Quality filtering was performed using the FASTX-Toolkit (v0.0.13.2; fastq_quality_filter –Q33 –q 30 –p 50). The quality filtered and trimmed reads were then digitally normalised [36]. Once all filtering was completed, reads from all stages were combined and the transcriptome was assembled using the Trinity package (v2013–02-25) [37]. Partial and complete open reading frames (ORFs) with a minimum length of 100 amino acids were predicted using the TransDecoder (version rel16JAN2014) script. Bacterial contaminants were obtained using mpiBlast (v.1.6) [65] with e-value 1E-20 and crosschecked with hits obtained against UniProtKB-SwissProt with the same e-value. Searches with mpiBlast were run on the Legion HPC cluster at UCL on at least 40 cores. Sequences with higher similarity to the bacterial database were removed from the dataset. The cleaned ORF dataset represents the reference transcriptome (RefTr). All reads were deposited in the NCBI Short Read Archive (SRA) under accession numbers SRR4436669–SRR4436674.

#### Preparation of other datasets

Transcriptome sequence data from *A. mediterranea* was obtained by the Elphick lab at Queen Mary University of London, as reported previously [42, 66]. To obtain a complete picture of coding sequences from *P. miniata*, we combined both genomic derived coding sequences and transcriptome sequences from http://echinobase.org [67].

#### Quality assessment

Completeness of our transcriptome was estimated using CEGMA (v2.5) [68] and BUSCO (v3.0) [69]. Full-length distributions were estimated by considering all unique hits determined by BLASTx (1e-20) against the UniProtKB-SwissProt database and application of scripts included within the Trinity application.

#### Annotation

All BLAST [70] searches were performed using a local NCBI-BLAST (v2.2.25) with e-value of 1e-6. The RefTr was annotated against the sea urchin *S. purpuratus* transcriptome sequences and against the UniProtKB-SwissProt database. One directional BLAST identified presumed homologs and reciprocal BLAST identified presumed orthologs. Gene ontology classification was performed based on a previous sea urchin-specific classification [21]. For consistency purposes sequences obtained for the sea star *P. miniata* (http://www.echinobase.org/Echinobase/) and the crinoid *A. mediterranea* raw sequences [42] were annotated using the same combination of one-directional and reciprocal BLAST (e-value 1e-6) against the sea urchin transcriptome database.

#### Abundance estimation

The quality filtered trimmed reads were re-aligned on the reference transcriptome using bowtie (v0.12.9) [71] with parameters set as in RSEM [72]. Reads for chemically perturbed samples were filtered out. The bowtie output was loaded into CORSET in order to obtain counts for clusters of contigs that shared reads, rather than individual contigs [46]. This is equivalent to a potential “gene” count adding up all “isoform” counts. Normalization by internal standard was performed as follows: First, individual clusters were normalised by their peak of expression in the time-course data (9, 18, 27 and 39 hpf); then, for each cluster the standard deviation was calculated and clusters with standard deviation below 0.01 were chosen as internal standard; and finally, an average of these clusters was used as normalization factor and each cluster was divided by this normalization factor and multiplied by 1,000,000. All downstream analysis was performed using customised R and bash scripts. In order to make statements about annotation content in the individual clusters, the most frequent annotations for each expression cluster were considered.

#### Expression clustering of time-series data

To sort expression clusters by their individual trajectories we applied the fuzzy clustering algorithm [48]. We used 27 fuzzy clusters, based on the assumption that between four sampled time points the expression either increased, decreased or did not change giving 3^3^ (27) possible paths for each trajectory. Note here the difference between a fuzzy cluster and an expression cluster: a fuzzy cluster describes a group of expression clusters that share similar trajectories over time. Since fuzzy clustering does not allocate each transcript always to the same cluster, we re-iterated this algorithm 100 times to find for each expression cluster the most probable fuzzy cluster membership.

#### Estimation of phylogenetic trees

Homologous sequences of Msp130 genes were selected from OMA output and used as input to build a HMM model using HMM 3.1 (http://hmmer.org, version 3.1b). Protein databases of seven selected species were used to aggregate contigs with a conserved HMM domain. The determined contigs were filtered from redundant and small sequences with length below 100 amino acids. For the msp130 alignment specifically, additional sequences were obtained from *Ophiothrix spiculata* and *Lytechinus variegatus*. The sequences were aligned using PRANK [73]. The resulting alignment was then inspected using sea view and trees were estimated using PhyML v3.1 [74] and PhyloBayes MPI 1.6j [75]. Topological differences are displayed using http://phylo.io [76].

## Additional files

Additional file 1: Supplementary **Figures S1** to **S12**. (PDF 3084 kb)

Additional file 2: Supplementary **Tables S1** to **S7**. (DOCX 24 kb)

Additional file 3: All vs all search for 901 skeletogenic genes. This file includes the detailed results of Fig. 2 and Additional file 1: Figure S5 to show in detail which genes are parts of the different overlaps between the four species. (XLSX 197 kb)

Additional file 4: Stable genes used for normalization. This file includes a list of genes that have a standard deviation smaller than 0.01 and whose average was used as normalization for the expression of the residual genes. (XLSX 70 kb)

Additional file 5: Details on fuzzy clustering. This file includes the data for Fig. 5. It shows the classification of TFs of *A. filiformis* and *S. purpuratus* into the four modes of expression. (XLSX 69 kb)

## Abbreviations

Afi: *Amphiura filiformis*
Ame: *Antedon mediterranea*
EC: Expression cluster
FC: Fuzzy cluster
GRN: Gene regulatory network
Mya: Million years ago
OMA: Orthology matrix algorithm
Pmi: *Patiria miniata*
RefTr: Reference transcriptome
sm: Skeletogenic mesoderm
SNP: Single nucleotide polymorphism
Spu: *Strongylocentrotus purpuratus*
SUFCs: Sea urchin functional classes
TF: Transcription factor
