# Supplementary material for: Developmental transcriptomics of the brittle star Amphiura filiformis reveals gene regulatory network rewiring in echinoderm larval skeleton evolution
Source: Genome Biol. 2018 Feb 28;19:26. doi: 10.1186/s13059-018-1402-8 (PMC5831733; doi:10.1186/s13059-018-1402-8)
Supplement: Supplementary file 1 — Supplementary Figures S1 to S12. (PDF 3084 kb) [file 13059_2018_1402_MOESM1_ESM.pdf]

Supplementary Figures

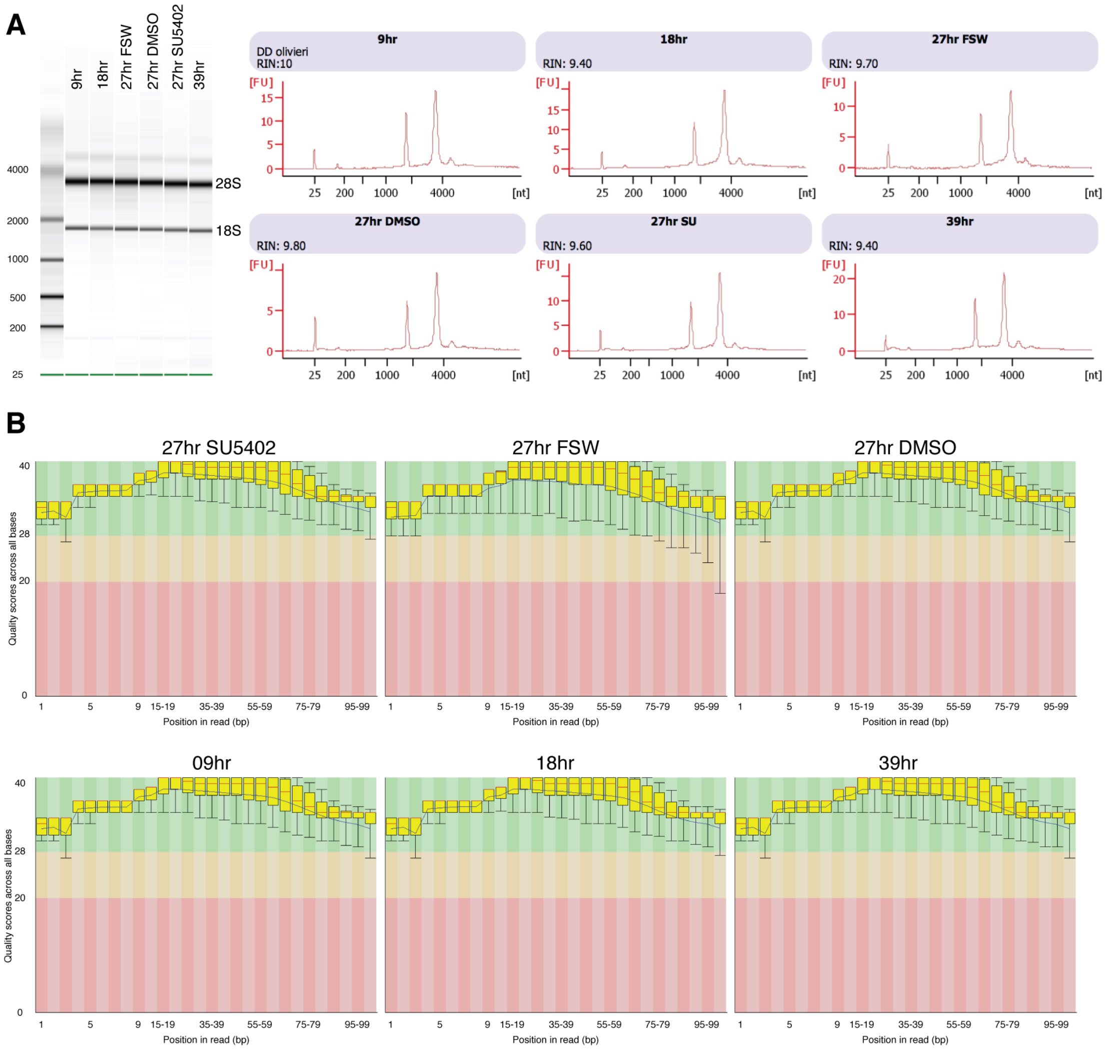

Figure S1. **High quality RNA samples resulted in high quality reads.** (A) Bioanalyser image and electropherogram of six samples sent for sequencing. The ribosomal RNAs 18S and 28S show little to no degradation. The Y-axis indicates the fluorescence (FU) and the x-axis indicates the molecular weight of the RNA in nucleotides (nt). (B) All samples have high quality reads (average of basepair quality within green area), as determined by FastQC.

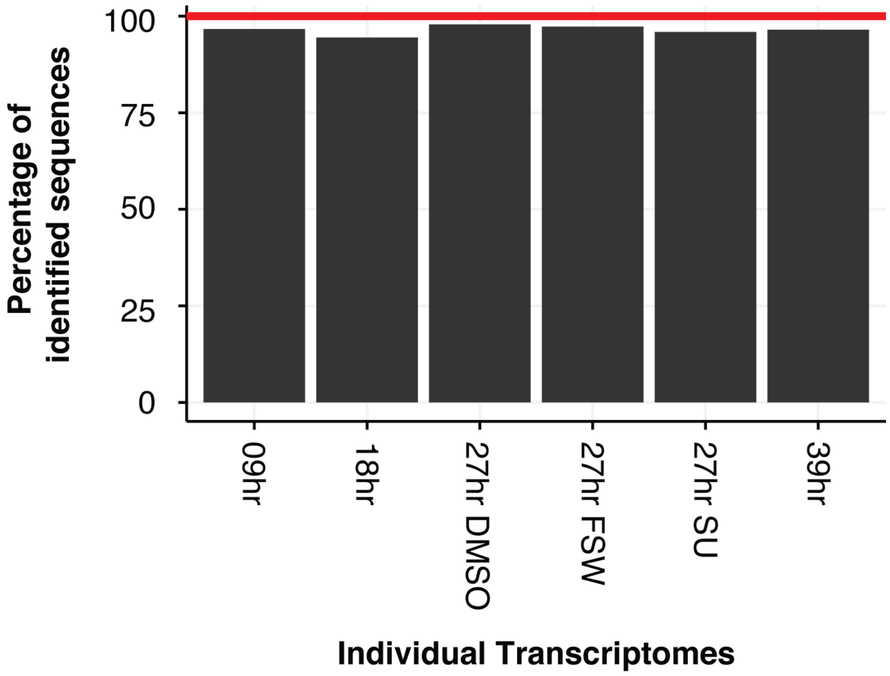

Figure S2. **Digital normalization does not bias our samples.** Blastn (e-value: 1E-20) search of individually assembled transcriptomes against the combined assembled transcriptome with digital normalization step (IT) shows preservation of more than 94% of contigs of each individual transcriptome in the IT.

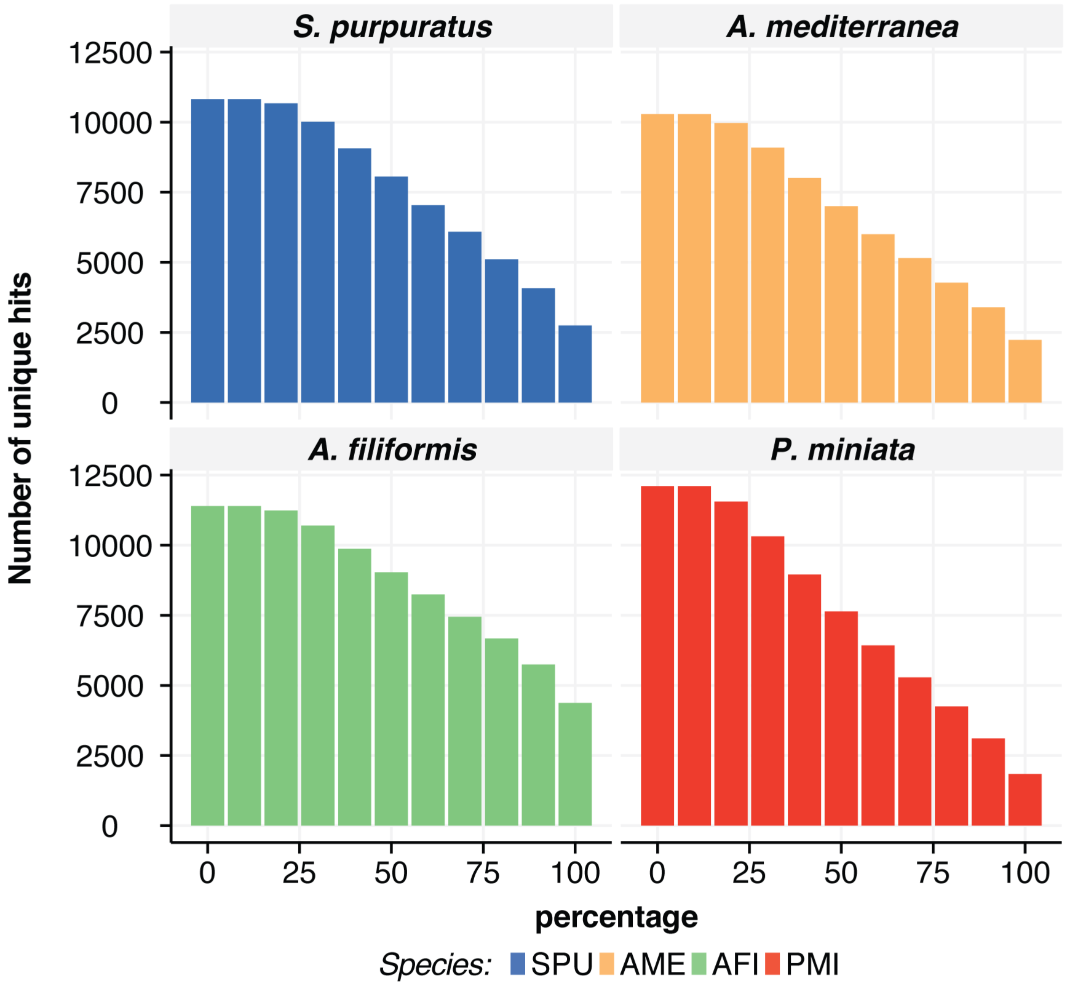

Figure S3. **Full length contig analysis for four representative species of the Echinodermata**. The brittle star AFI has the best alignment coverage with 4,373 contigs for which each contig has an alignment that is covered by more than 90% of a gene identified in the SwissPort DB. This is followed by sea urchin SPU with 2,748 contigs, then by sea lily AME with 2,234 contigs and by starfish PMI with 1,838 contigs. Values were obtained by blastx of each dataset against the SwissProt DB (e-value: 1E-20) and estimation of number of unique hits per percentage of alignment coverage.

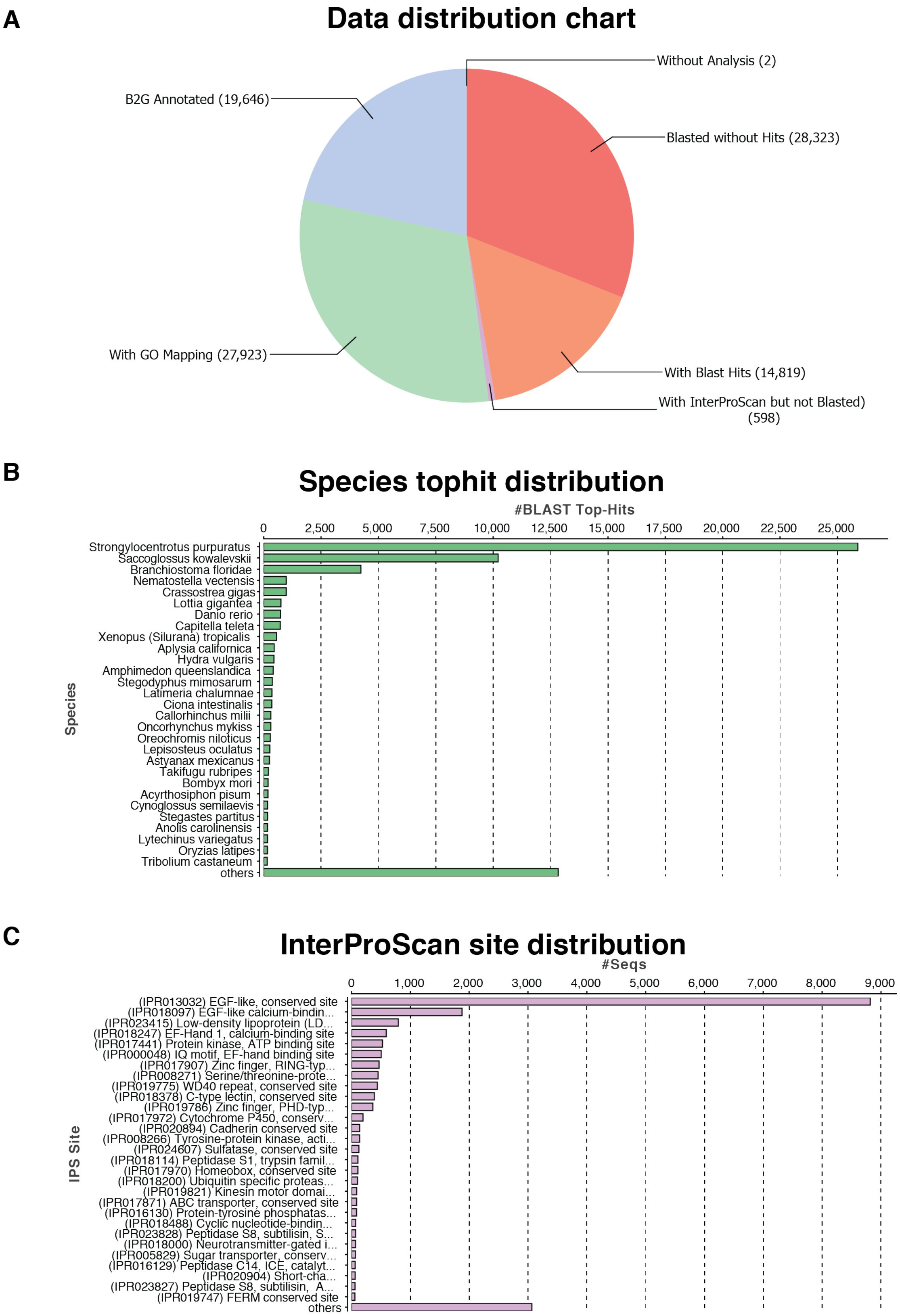

Figure S4. **Summary statistics of results obtained using Blast2GO.** (A) Piechart showing number of sequences and their individual annotation statistics. (B) Species tophit distribution showing that genes identified when blasting (e-value: 1E-3) against the non-redundant DB belonged to the sea urchin Strongylocentrotus purpuratus. (C) InterProScan site distribution

identifies a high representation of genes that have an EGF-like conserved site.

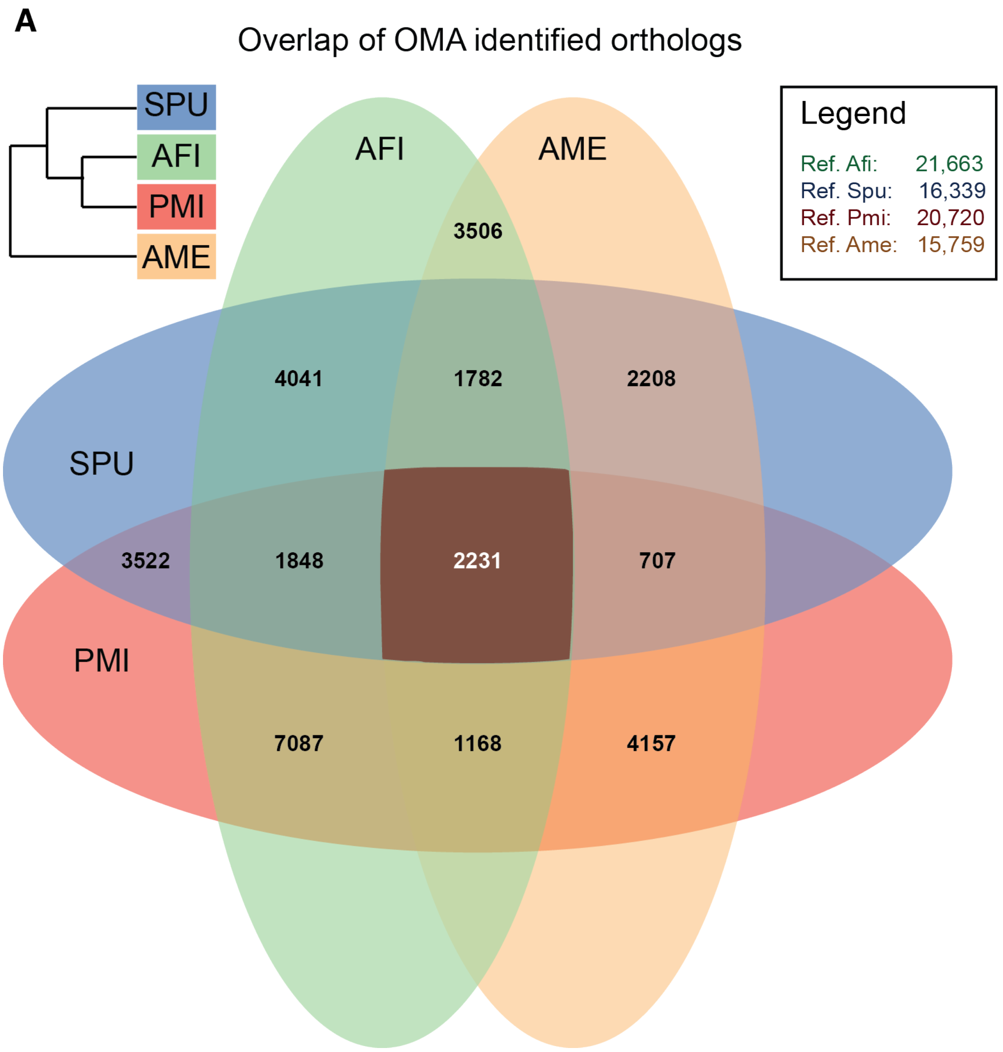

Figure S5. **Echinoderm gene overlap identified using the OMA orthology predication algorithm.** Venn diagram showing overlap of sea urchin found genes in the individual species using the OMA orthology prediction algortihm. Afi - *Amphiura filiformis*, Pmi - *Patiria miniata*, Ame - *Antedon mediter- ranea*, Spu - *Strongylocentrotus purpuratus*, Brown - Echinoderm Core (overlap of all four species).

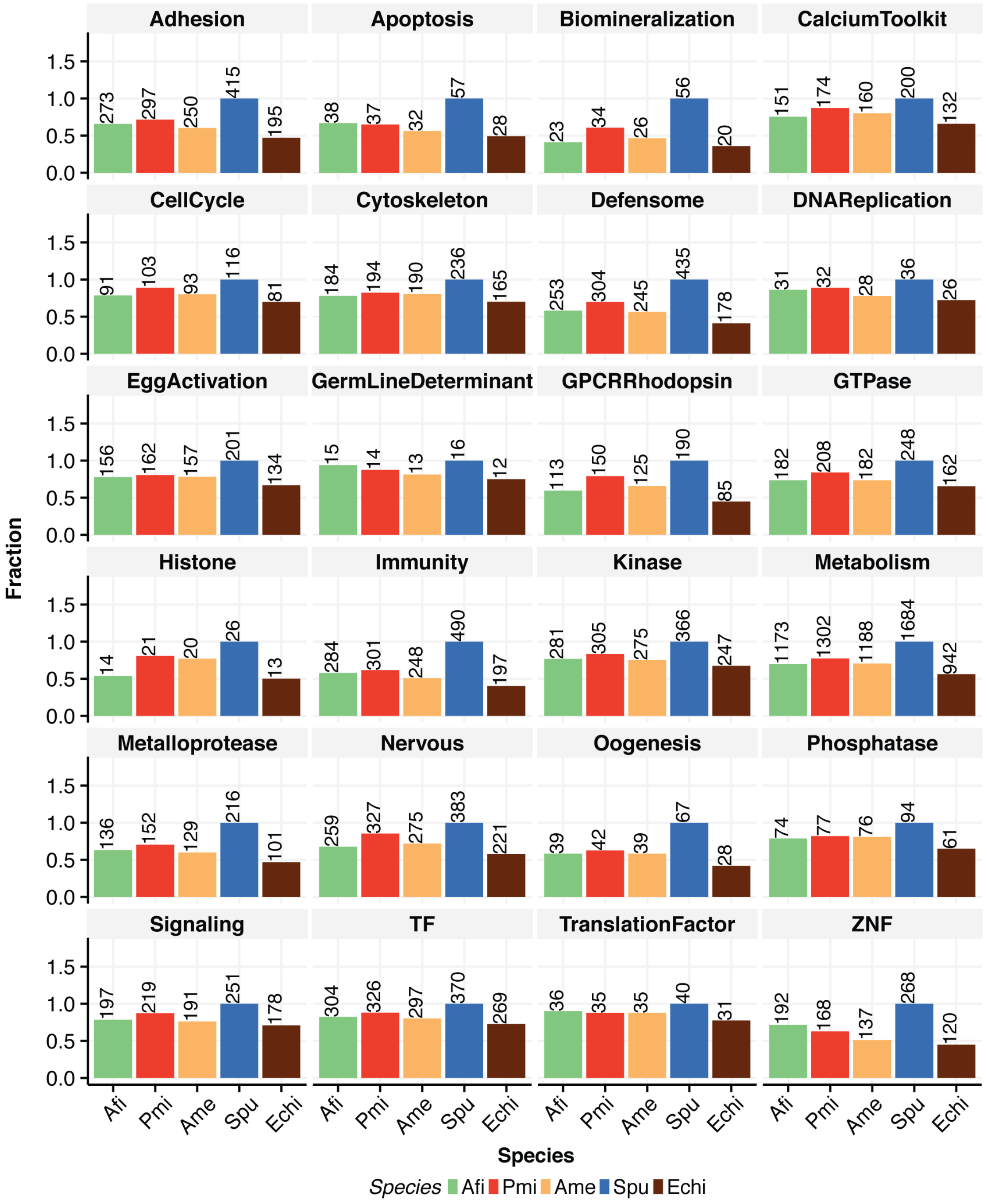

Figure S6. **Gene Ontology classification of echinoderms.** Annotated sequences were queried for sequences with assigned gene ontology classes established for sea urchin and numbers rep- resent the sum of sequences belonging to one of the 24 GO classes. Afi - *Amphiura filiformis*, Pmi - *Patiria miniata*, Ame - *Antedon mediterranea*, Spu - *Strongylocentrotus purpuratus*, Echi - Echinoderm Core (overlap of all four species).

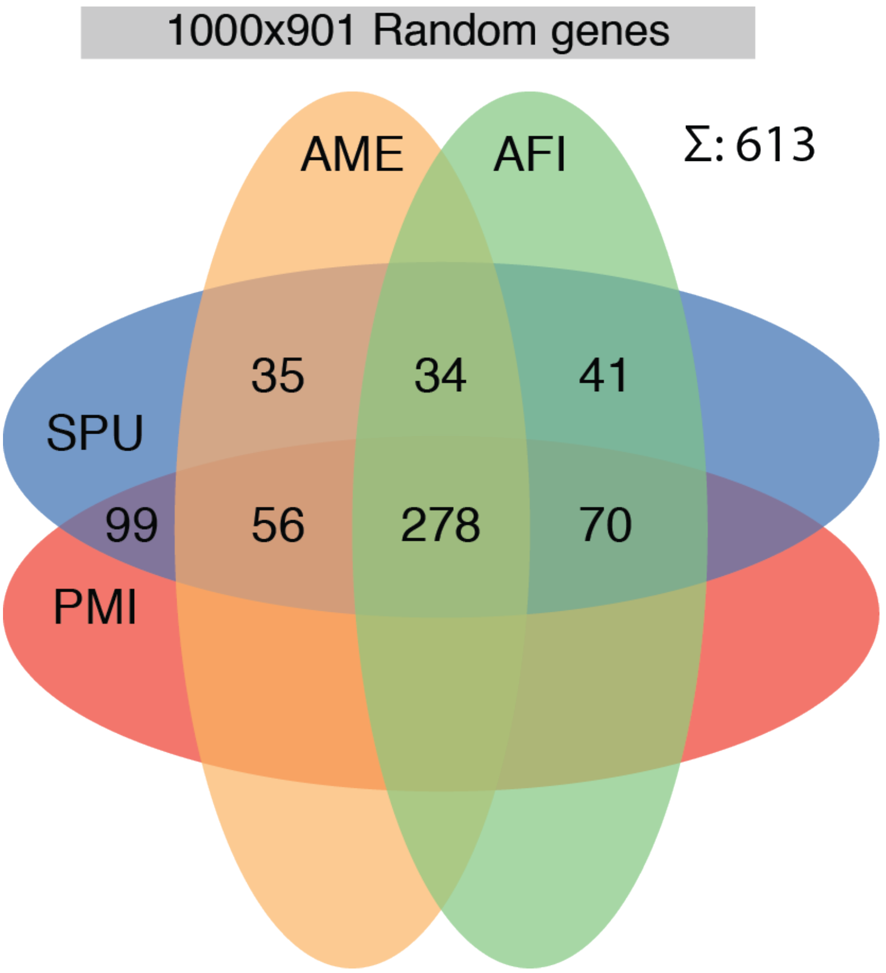

Figure S7 **Conservation of skeletogenic genes in echinoderms.** (A) Annotated species datasets were queried for a sea urchin list of skeletogenic genes or 1000 times random genes and overlaps were estimated. All species share 494 skeletogenic genes which represents a set of genes higher conserved than a randomly picked set of genes that share 278 in average (χ^2 proportion test: $p<0.001$).

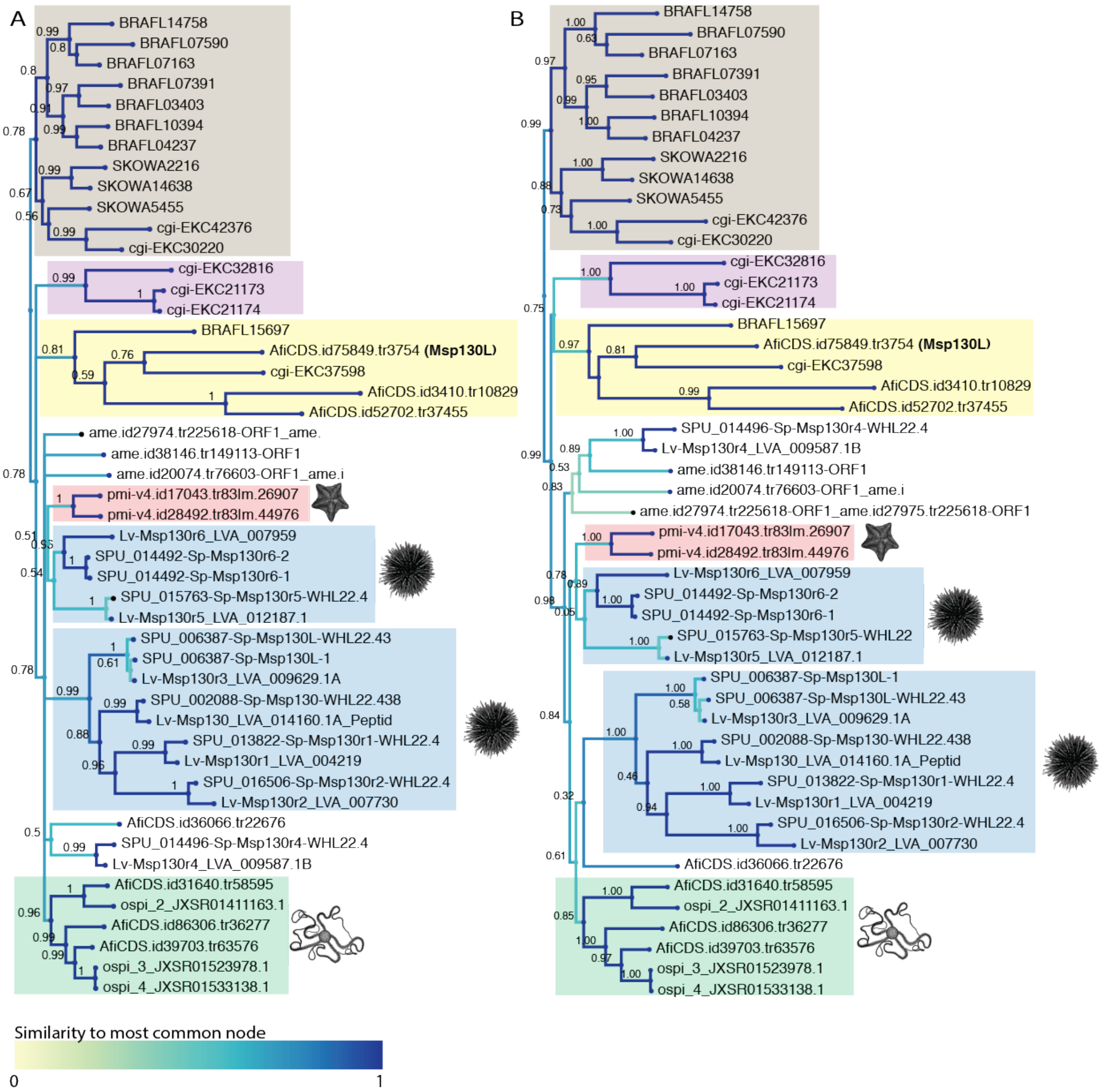

Figure S8. **Phylogeny of msp130 protein sequences supports independent duplication of these genes in the four main groups of echinoderms.** (A) Tree constructed using a maximum likelihood approach (PhyML). Branch support calculated using 100 bootstrap replicates. (B) Tree computed using a Bayesian approach (PhyloBayes). Posterior distribution values are shown, once the MaxDiff parameter converged to below 0.3. Topological differences between the two trees are displayed using http://phylo.io.

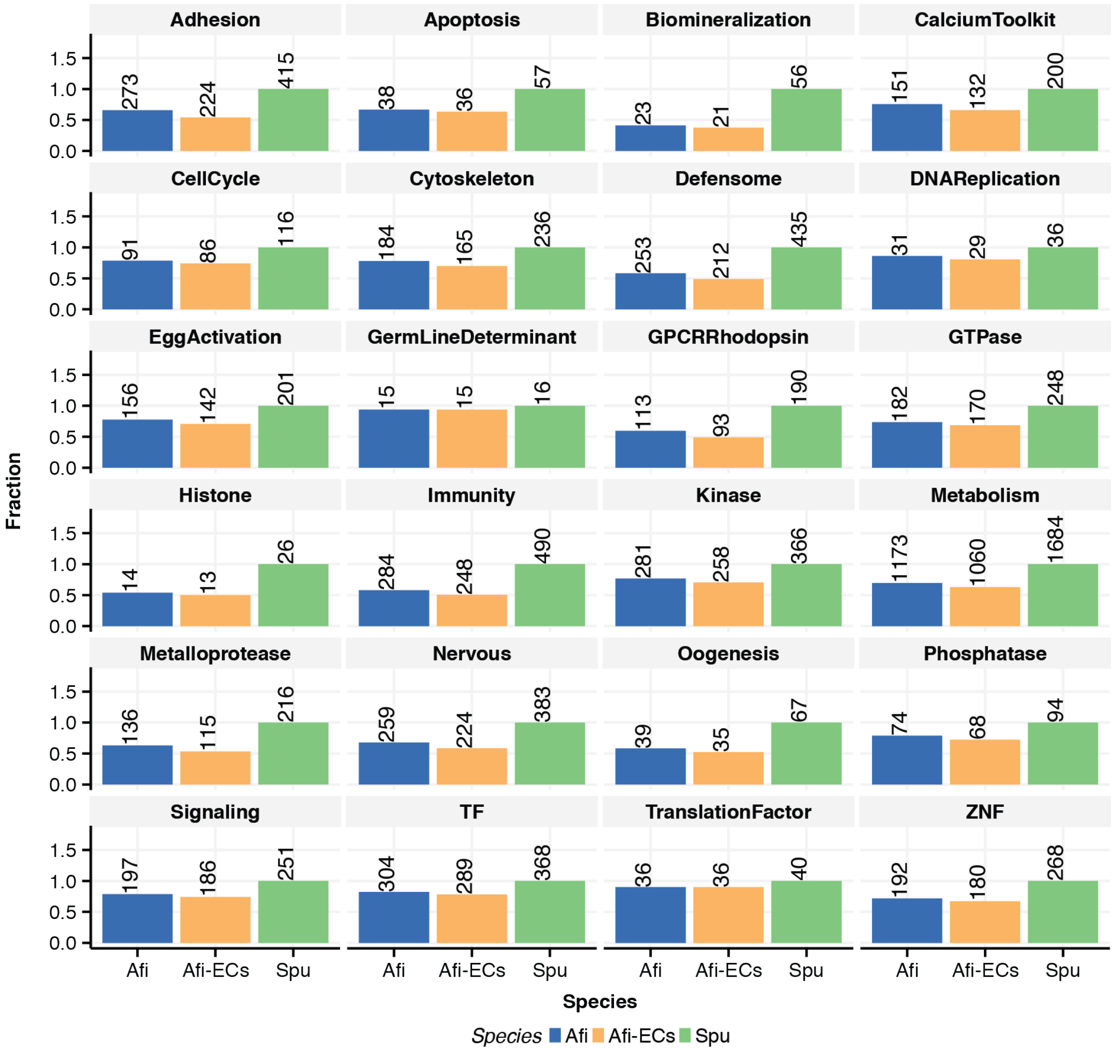

Figure S9. **Loss of genes grouped by ontology classes after grouping into ECs**. The reduction of ~91.000 contigs to ~39.000 expression clusters (ECs) showed little loss in individual gene families, thus still conserving a good representation of gene content and giving support for the CORSET contig clustering strategy.

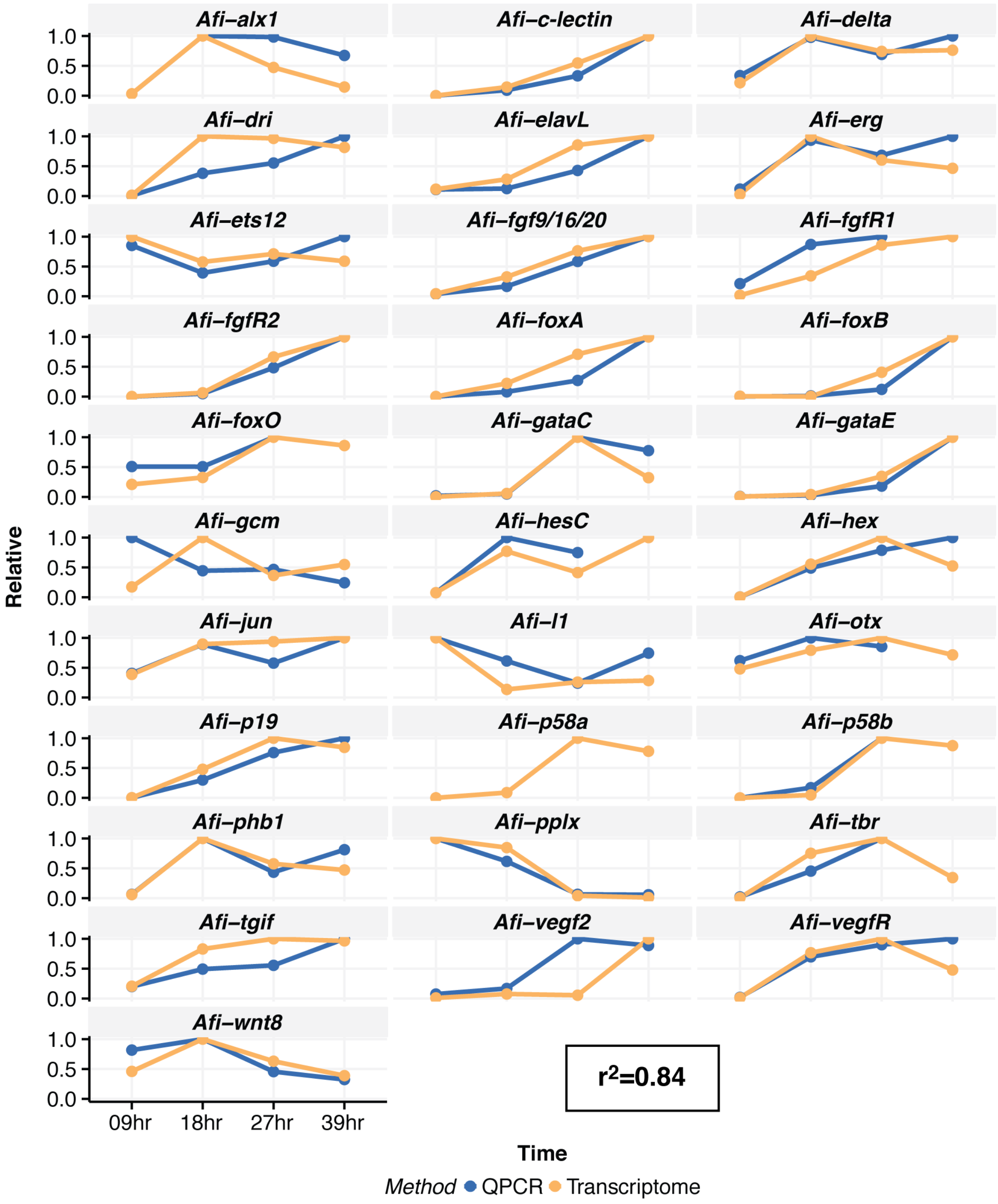

Figure S10. **Comparison of QPCR vs Transcriptome shows high correlation.** Comparison of normalised expression of QPCR and Transcriptome results in an average correlation r^2 =0.84. Low correlation is observed for *Afi-gcm*, however consistent with low absolute values for expression in both approaches.

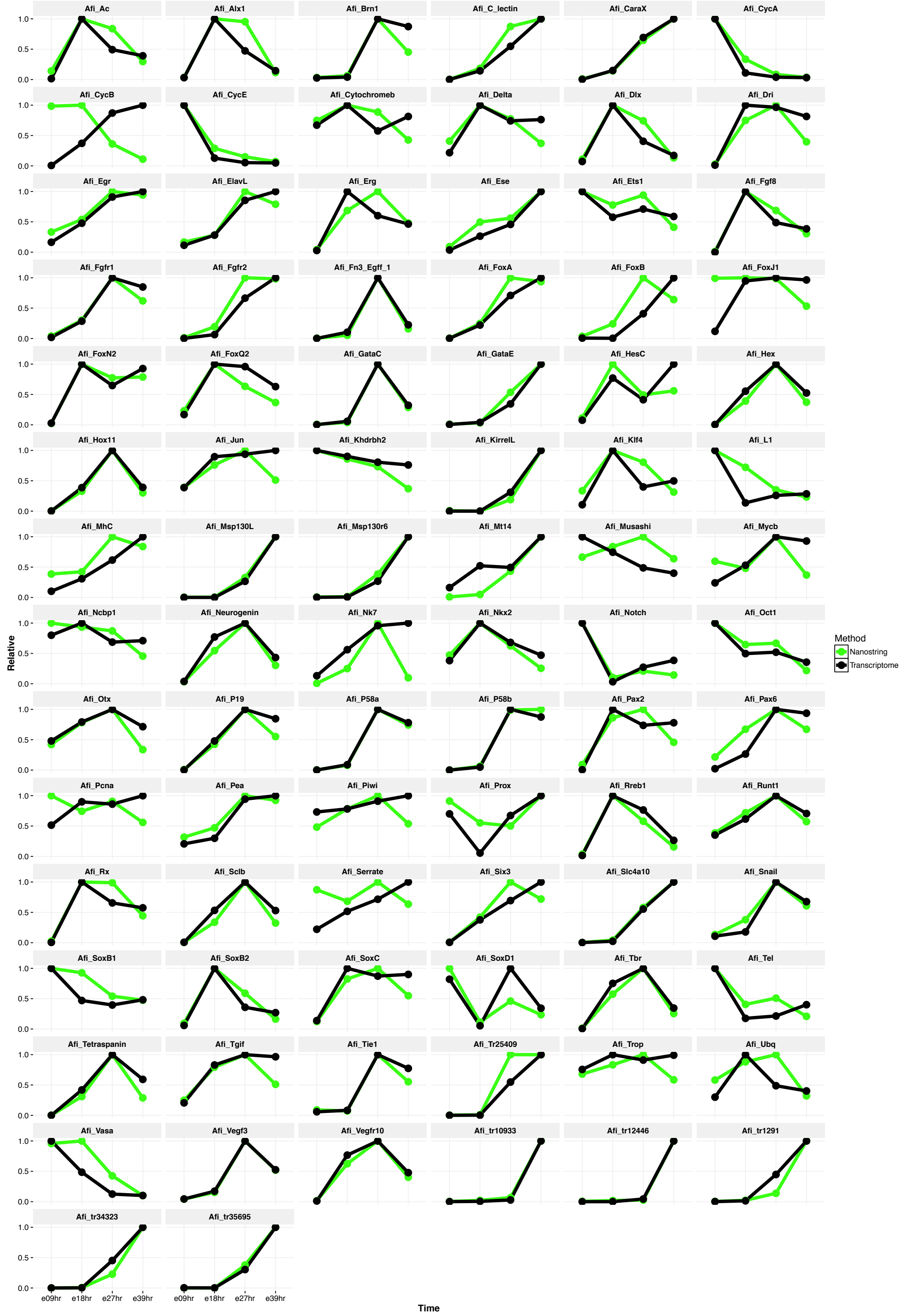

Figure S11. **Comparison of Nanostring vs Transcriptome shows high correlation.** Comparison of normalised expression of Nanostring and Transcriptome results in an average correlation r^2 =0.77.

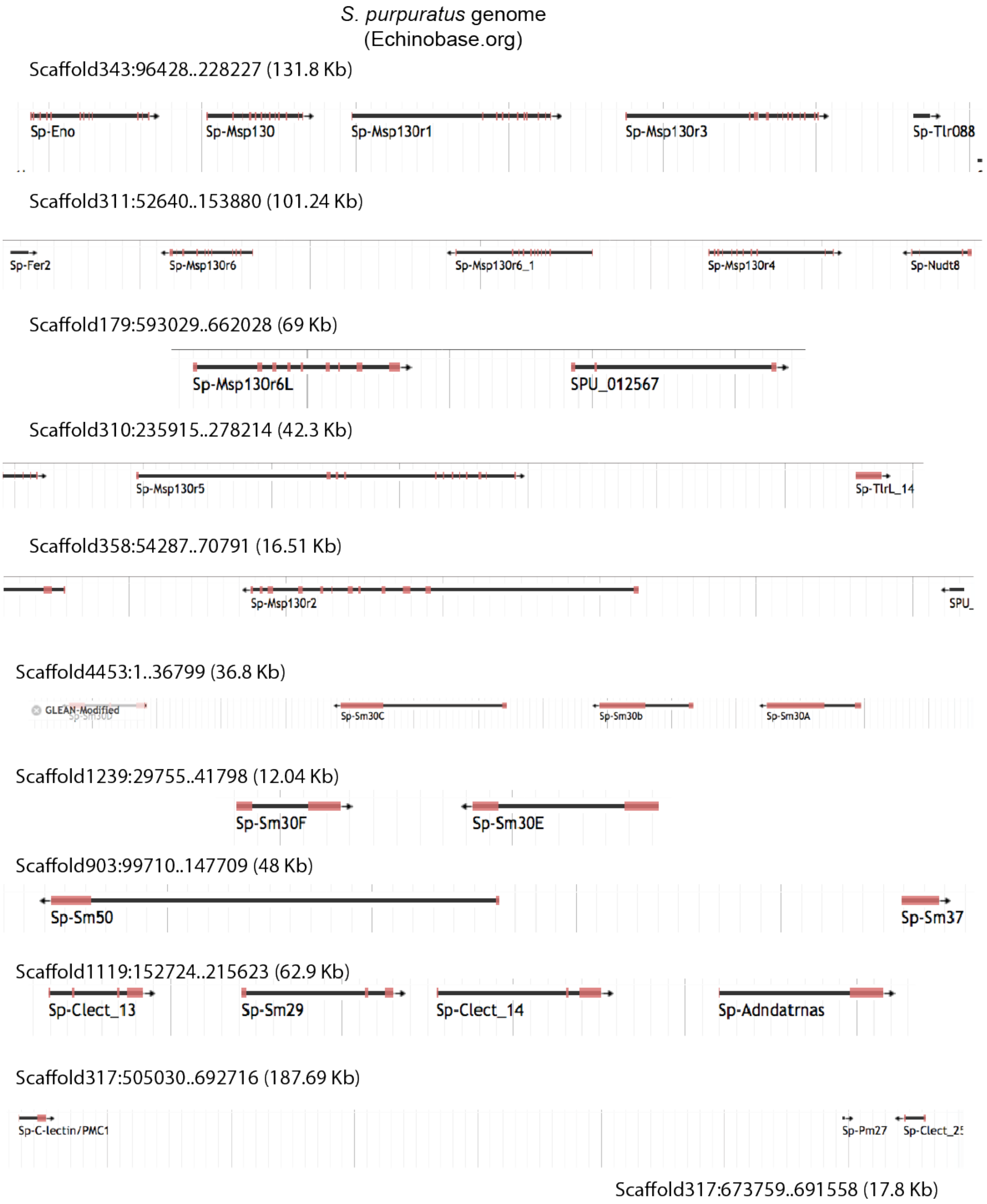

Figure S12. **Location of msp130 and sm genes on sea urchin scaffolds.** Msp130, msp130r1 and msp130r3 are adjacent to each other on the genome. Also msp130r6, msp130r6_1 and msp130r4 are adjacent. On the other hand msp130r6L, msp130r5 and msp130r2 are not in tandem. Similar for sm genes we observe the collocation of sm30C, sm30b, sm30a and sm30f with sm30e and sm50 with sm37 and clect_13, sm29 with clect_14. Only c-lection/PMC1 is not collocated with other sm genes.
