# Supplementary material for: Developmental transcriptomics of the brittle star Amphiura filiformis reveals gene regulatory network rewiring in echinoderm larval skeleton evolution
Source: Genome Biol. 2018 Feb 28;19:26. doi: 10.1186/s13059-018-1402-8 (PMC5831733; doi:10.1186/s13059-018-1402-8)
Supplement: Supplementary file 2 — Supplementary Tables S1 to S7. (DOCX 24 kb) [file 13059_2018_1402_MOESM2_ESM.docx]

**Table S1.** Summary statistics of sequencing runs and read processing

| **Sample** | **# reads** | **After trimming** | **After digital normalization** | **Percentage used for assembly** |
| --- | --- | --- | --- | --- |
| **09hr** | 110,346,804 | 109,378,446 | 38,316,233 | 33.67 |
| **18hr** | 117,679,784 | 113,488,210 | 39,997,943 | 32.98 |
| **27hr FSW** | 126,343,356 | 125,426,980 | 41,364,607 | 31.85 |
| **27hr DMSO** | 124,834,432 | 122,436,458 | 40,916,321 | 31.87 |
| **27hr SU** | 107,636,502 | 105,276,888 | 39,610,310 | 35.78 |
| **39hr** | 128,647,022 | 127,546,868 | 44,834,020 | 33.87 |
|  |  |  |  |  |
| **Total** | 715,487,900 | 703,553,850 | **245,039,434** | 33.27 |

**Table S2.** Number of sequences with length larger than

| **Species** | **>1000bp** | **>2000bp** | **>3000bp** |
| --- | --- | --- | --- |
| *Strongylocentrotus purpuratus* | 22,596 | 15,392 | 10,425 |
| *Amphiura filiformis* | 25,637 | 9,096 | 3,795 |
| *Patiria miniata* | 21,671 | 5,453 | 1,912 |
| *Antedon mediterranea* | 26,884 | 10,452 | 4,891 |

**Table S3.** CEGMA output for all species used in this study

| **Species** | **# complete 248 CEGs** | **# partial 248 CEGs** |
| --- | --- | --- |
| *Strongylocentrotus purpuratus* | 209 | 246 |
| *Amphiura filiformis* | 205 | 238 |
| *Patiria miniata* | 193 | 240 |
| *Antedon mediterranea* | 221 | 246 |

**Table S4.** BUSCO output against 978 Metazoan genes

| **Species** | **Complete** | **Fragmented** | **Missing** |
| --- | --- | --- | --- |
| *Strongylocentrotus purpuratus* | 87.6% | 7.7% | 4.7% |
| *Amphiura filiformis* | 97.9% | 0.9% | 1.2% |
| *Patiria miniata* | 83.2% | 12.3% | 4.5% |
| *Antedon mediterranea* | 90.6% | 8.5% | 1.0% |

**Table S5.** Summary of biomineralization class after curation

| **Biomineralization** | ***Afi*** | ***Pmi*** | ***Ame*** | ***Spu*** |
| --- | --- | --- | --- | --- |
| **Carbonic Anhydrase** | 2 | 3 | 3 | 3 |
| **Collagen** | 10 | 15 | 9 | 15 |
| **Cyclophilin** | 7 | 7 | 9 | 11 |
| **MSP130** | 7 | 2 | 3 | 9 |
| **Spicule Matrix (SM)** | 1 | 1 | 0 | 14 |
| **Other** | 0 | 1 | 0 | 4 |
| **Total** | 27 | 29 | 24 | 56 |

**Table S6.** Cloning primers

| Gene | Forward | Reverse | Length |
| --- | --- | --- | --- |
| Afi-007098 | ATGGGAATGTAGCCGATGTG | TGACAAACTCTCTGACAGTCTGA | 743bp |
| Afi-msp130L | CGTCTTACTCGTACCAGCCT | CTACTCCTGCTGCTGTTCCT | 878bp |
| Afi-ttrspn_19 | GGCGCTCGATGGCTGTTC | GAGGCTGTTTCCGTAAATCTTGA | 716bp |
| Afi-c-lectin | AGCAGCAATGAAGGTCTGGT | AAGACTGGAAGAAAACAAGA | 1317bp |
| Afi-p58-a | CCGTTCGAAACTAAGCATCGT | AGGTACCAGCTTTACTCTTGTT | 600bp |
| Afi-slc4a10 | CGATCCCTACTCGGTTCCTC | TCGCAGTCTTCCATAGCGAT | 988bp |
| Afi-p58-b | TGCTAAAGGAGGTGCTAAGGA | AATTCCTCCTCCAGCTCGTC | 702bp |
| Afi-lrr/Igr_10 | TACGGCTTGGAGATCTGGAC | CGCAGATTCGGTAGTGCAAA | 1316bp |
| Afi-kirrelL | GGTGAAACCGCAACTCTGAA | TGTTGAGTTCGTATCTGCGC | 1647bp |
| Afi-cebpa | AGTGACGACATTGGTGGACT | AGGACACTGAGAAGCTGCTT | 749bp |
| Afi-adam/tsl6 | GATCCCGACGGTGGTAGTAG | GTCCTGGTACCACTTCCACA | 800bp |
| Afi-mt14-mmpL5 | ATCAAGAGTCCTAGGTGCGG | GTTGGTTGGTGTATTCGGTGT | 606bp |
| Afi-picalmL | AACACACAATTACCGGCTCC | CCTGCCTCGTGATATCCAGA | 1500bp |

**Table S7.** Sea urchin SM and Msp130 clusters on scaffolds

| **Scaffold coordinates**  **(kb)** | ***S. purpuratus* genes** | **SPU number** | **Reference** |
| --- | --- | --- | --- |
| Scaffold343: 96428-228227 (131.8 Kb) | Sp-Msp130  Sp-Msp130r1  Sp-Msp130r3 | SPU_002088  SPU_013822  SPU_013823 | Livingston et al 2006 |
| Scaffold311: 52640-153880 (101.24 Kb) | Sp-Msp130r6  Sp-Msp130r6_1 Sp-Msp130r4 | SPU_014492  SPU_031101  SPU_014496 | Livingston et al 2006 |
| Scaffold179: 593029-662028 (69 Kb) | Sp-Msp130r6L | SPU_015326 | Livingston et al 2006 |
| Scaffold310: 235915-278214 (42.3 Kb) | Sp-Msp130r5 | SPU_015763 | Livingston et al 2006 |
| Scaffold358: 54287-70791 (16.51 Kb) | Sp-Msp130r2 | SPU_016506 | Livingston et al 2006 |
| Scaffold4453: 1-36799  (36.8 Kb) | Sp-Sm30A  Sp-Sm30B  Sp-Sm30C  Sp-Sm30D | SPU_000825  SPU_000826  SPU_000827  SPU_000828 | Livingston et al 2006 |
| Scaffold903: 99710-147709 (48 Kb) | Sp-Sm50  Sp-Sm37 | SPU_018811  SPU_018813 | Livingston et al 2006 |
| Scaffold1119: 152724-215623  (62.9 Kb) | Sp-Clect_13  Sp-Sm29  Sp-Clect14  Sp-Adndatrnas | SPU_005989  SPU_005990  SPU_005991  SPU_005992 | Livingston et al 2006 |
| Scaffold317: 505030-692716 (187.69 Kb) | Sp-C-lectin/PMC1  Sp-Pm27  Sp-Clect25 | SPU_027906  SPU_030147  SPU_011163 | Livingston et al 2006 |
| 9 scaffolds | 22 genes total |  |  |
